# Genetic susceptibility, elevated blood pressure, and risk of atrial fibrillation: a Mendelian randomization study

**DOI:** 10.1186/s13073-021-00849-3

**Published:** 2021-03-04

**Authors:** Milad Nazarzadeh, Ana-Catarina Pinho-Gomes, Zeinab Bidel, Dexter Canoy, Abbas Dehghan, Karl Smith Byrne, Derrick A. Bennett, George Davey Smith, Kazem Rahimi

**Affiliations:** 1 Deep Medicine, Oxford Martin School, University of Oxford, 1st Floor, Hayes House, 75 George Street, Oxford, OX1 2BQ UK; 2 Nuffield Department of Women’s and Reproductive Health, University of Oxford, Oxford, UK; 3 NIHR Oxford Biomedical Research Centre, Oxford University Hospitals NHS Foundation Trust, Oxford, UK; 4 Department of Biostatistics and Epidemiology, School of Public Health, Imperial College London, London, UK; 5 Clinical Trial Service Unit and Epidemiological Studies Unit, Nuffield Department of Population Health, University of Oxford, Oxford, UK; 6 MRC Integrative Epidemiology Unit, University of Bristol, Bristol, UK

**Keywords:** Blood pressure, Atrial fibrillation, Mendelian randomization

## Abstract

**Background:**

Whether elevated blood pressure (BP) is a modifiable risk factor for atrial fibrillation (AF) is not established. We tested (1) whether the association between BP and risk of AF is causal, (2) whether it varies according to individual’s genetic susceptibility for AF, and (3) the extent to which specific BP-lowering drugs are expected to reduce this risk.

**Methods:**

First, causality of association was assessed through two-sample Mendelian randomization, using data from two independent genome-wide association studies that included a population of one million Europeans in total. Second, the UK Biobank data of 329,237 participants at baseline was used to study the effect of BP on AF according to genetic susceptibility of developing AF. Third, a possible treatment effect with major BP-lowering drug classes on AF risk was predicted through genetic variants in genes encode the therapeutic targets of each drug class. Estimated drug effects were compared with effects on incident coronary heart disease, for which direct trial evidence exists.

**Results:**

The two-sample Mendelian randomization analysis indicated that, on average, exposure to a higher systolic BP increased the risk of AF by 19% (odds ratio per each 10-mmHg [OR] 1.19 [1.12 to 1.27]). This association was replicated in the UK biobank using individual participant data. However, in a further genetic risk-stratified analysis, there was evidence for a linear gradient in the relative effects of systolic BP on AF; while there was no conclusive evidence of an effect in those with low genetic risk, a strong effect was observed among those with high genetic susceptibility for AF. The comparison of predicted treatment effects using genetic proxies for three main drug classes (angiotensin-converting enzyme inhibitors, beta-blockers, and calcium channel blockers) suggested similar average effects for the prevention of atrial fibrillation and coronary heart disease.

**Conclusions:**

The effect of elevated BP on the risk of AF is likely to be causal, suggesting that BP-lowering treatment may be effective in AF prevention. However, average effects masked clinically important variations, with a more pronounced effect in individuals with high genetic susceptibility risk for AF.

**Supplementary Information:**

The online version contains supplementary material available at 10.1186/s13073-021-00849-3.

## Background

Atrial fibrillation (AF) is the most common clinically important cardiac arrhythmia and its incidence and prevalence are on the rise worldwide [1]. AF is associated with an increased risk of fatal and nonfatal cardiovascular events [2], and its associated care and management impose substantial burden on healthcare systems [3].

Evidence on how to effectively prevent AF, thus far, has been limited. Observational studies have estimated that a 20-mmHg higher systolic blood pressure (SBP) was associated with a higher risk of AF [hazard ratio (HR) 1.21, 95% confidence interval (CI) 1.19 to 1.22] [2]. However, randomized controlled trials (RCTs) and their meta-analyses have shown no compelling evidence that blood pressure (BP)-lowering treatment reduces the risk of AF [4–6]. This apparent discrepancy between observational and randomized evidence may be related to limitations of observational studies which are prone to reverse causality and residual confounding. It is also possible that randomized trials have been underpowered to detect an effect size that, based on observational evidence, seems more modest than what has been reported for atherosclerotic events like stroke or myocardial infarction. Besides, a meta-analysis of RCTs investigating the effect of BP-lowering treatment on the risk of AF found observable heterogeneity in effect sizes that were partially explained by differences in trial-level baseline risk for AF [6]. This has raised the hypothesis that the overall weak or lack of apparent treatment effects could be masking stronger effects in high-risk individuals in whom BP-lowering could have benefits in reducing their future risk of AF.

Recent advances in genome-wide association studies (GWAS) have revealed a large number of genetic variants that play an important role in the etiology of AF [7]. These, together with the increasing availability of genetic data from large-scale biobanks, provide an opportunity to utilize Mendelian randomization (MR) to take the evidence from previous observational epidemiological studies and clinical trials further. In this multi-step study, the main aims were to investigate the causal association between elevated SBP and risk of AF and to assess the extent to which this association varies according to genetic susceptibility for AF. Furthermore, to inform clinical decision-making, we also aimed to predict the effects of BP-lowering treatment for major BP-lowering drug classes. Results of this study have been published as an abstract at the European Society of Cardiology (ESC) congress 2020 [8].

## Methods

**Fig. 1:**
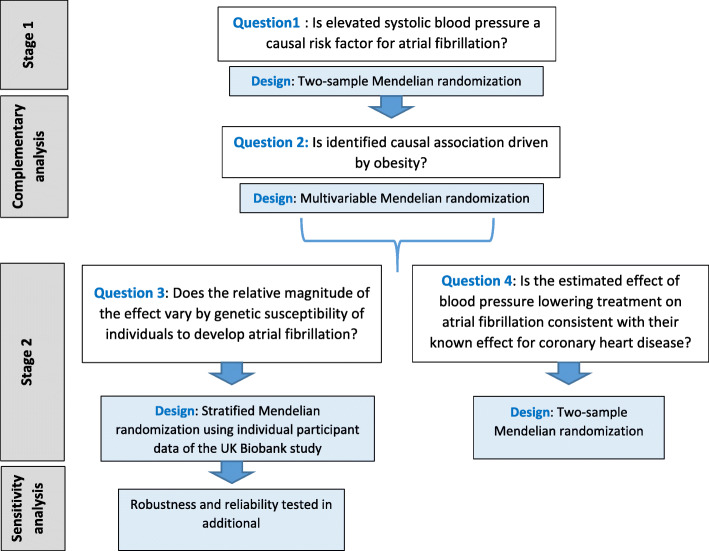
General structure of study including research questions and the specific research designs used to answer the stated questions

We employed different MR techniques to investigate several complementary research questions (Fig. 1 and Additional file 1: Supplementary methods). In general, MR takes advantage of the similarities between the random allocation of genetic variants at fertilization and that of random allocation of interventions in RCTs [9–11]. The different techniques and stages of analyses are described in more detail herein.

### Two-sample Mendelian randomization

#### Data for exposure

Our main exposure was genetically determined SBP as an instrumental variable. This was estimated from genetic variants with minor allele frequency > 0.01 that was independently associated (linkage disequilibrium r^2^ < 0.05) with SBP at *p* < 5 × 10^−8^. Overall, 251 genetic variants were used, all with imputation quality > 0.9 that have been shown to be associated with SBP in a GWAS meta-analysis including over one million European population (Additional file 2: Table S1 and Additional file 3: Figure S1) [12]. Because of partial overlap between the GWAS selected for exposure and outcome (UK biobank contributing to both) [7, 12], and to avoid weak instrument bias [13], we extracted the corresponding beta coefficients and standard errors from the International Consortium for Blood Pressure GWAS (ICBP), which did not include the UK biobank [14] and therefore provided a non-overlapping sample. ICBP is a GWAS meta-analysis including about 200,000 European population, and its estimations were adjusted for sex, age, age-squared, body mass index (BMI), within-cohort stratification, and also for BP-lowering medications use [14]. The ICBP analyses were conducted using linear regression model and combined across studies using inverse-variance weighted meta-analysis [14].

#### Data for outcome

The main outcome of interest was AF. We retrieved corresponding summary statistics from the largest and recently published GWAS meta-analysis, which included a total of 60,620 AF cases and 970,216 controls of European ancestry [7]. The GWAS included all genetic variants that were available for meta-analysis in the following studies: The Nord-Trøndelag Health Study (HUNT), DeCODE (The Icelandic AF population), The Michigan Genomics Initiative (MGI), The DiscovEHR collaboration cohort, AFGen Consortium, and UK Biobank. Estimations from each GWAS were combined using fixed-effects inverse-variance weighted meta-analysis with adjustment for population stratification within each cohort study [7].

We tested the validity of the instrumental variable by examining the causal association between SBP and positive outcomes including coronary heart disease, myocardial infarction, and ischemic stroke. For this analysis, we used two-sample MR using MR-base analytical platform [15]. We used the same genetic variants for SBP, but the variants-outcome association was extracted from independent GWAS studies [16, 17].

#### Analysis of two-sample Mendelian randomization

The summary data were harmonized before conducting the statistical analysis as recommended by Fortier et al. [13, 18]. We estimated the causal effects using a random-effect inverse variance weighted and applied various sensitivity analysis methods of two-sample MR including weighted median, MR pleiotropy residual sum and outlier [MR-PRESSO], Mendelian randomization analysis using mixture models (MRMix), Robust Adjusted Profile Score (RAPS), and MR-Egger and mode-based estimate (MBE) (Additional file 1: Supplementary methods). We examined the heterogeneity of the estimates by using a scatter plot and applying Cochran’s *Q* test [19]. We also assessed the probable directional pleiotropy using a funnel plot similar to that being used to assess for publication bias in meta-analysis [19]. A leave-one-out sensitivity analysis was conducted by removing a single variant from the analysis in turn. The fluctuation of the estimates in response to excluding each variant reflects the possibility of an outlier variant in the causal estimation. The “MendelianRandomization” and “TwoSampleMR” packages for R were used to implement the MR analyses [15, 20].

#### Complementary analysis

All the GWAS studies with SBP as phenotype routinely adjust for the effect of BMI [14, 21]. Using the estimates from BMI-adjusted GWAS to conduct an MR study could introduce collider bias. Therefore, we explored whether the identified causal association is driven by BMI using unadjusted BP estimations and by including BMI as a phenotype in multivariable MR. The UK Biobank dataset was used to derive the unadjusted estimates [22]. We used multivariable MR through inverse-variance weighted method [23, 24] to calculate adjusted versus unadjusted causal estimations.

### Stratified Mendelian randomization

To assess the stratified effect of SBP on AF by genetic susceptibility of developing AF, we followed a one-sample MR framework using individual participant data from the UK Biobank. The genetic variants used in this analysis were extracted from the UK Biobank imputation dataset (version 3). The details of inclusion and exclusion criteria are described in Additional file 1: Supplementary methods. In brief, 329,237 white British individuals with valid genetic data and complete BP measurements were included in this analysis. To determine genetic susceptibility of AF for each participant, we generated a genetic risk score using 102 genetic variants with minor allele frequency > 0.01 that were independently (*r*^2^ < 0.05) associated with AF at *p* < 5 × 10^−8^ in the last published GWAS in the European population (Additional file 1: Supplementary methods, Additional file 3: Figure S2, and Additional file 2: Table S2) [7]. Four categories were developed including the low, mild, moderate, and high genetic risk of AF (Additional file 3: Figure S3). We conducted a one-sample MR overall and then stratified by four categories of genetic susceptibility for AF to investigate whether the magnitude of the causal effect varied according to the category of genetic susceptibility. Details of statistical analysis and diagnostic codes are described in Additional file 1: Supplementary methods. To test whether the effect of SBP on the risk of AF was modified by genetic susceptibility for AF, we applied a likelihood ratio test for heterogeneity of trends comparing two multivariable logistic regression models.

#### Sensitivity analyses

The validity of the instrumental variable was checked through positive control analysis using coronary heart disease, myocardial infarction, and stroke as positive outcomes. We further performed a sensitivity analysis excluding all cases of coronary heart disease, heart failure, and valvular heart disease in the UK biobank to check that the observed associations between SBP and AF are not driven by the well-known association of SBP with these outcomes [25]. We performed a sensitivity analysis to investigate the possible impact of AF case definition on the associations by restricting the analysis to various components of AF definition (primary or secondary causes of hospitalization, or self-reported diagnosis). We adjusted the association for possible confounders (BMI, alcohol intake, smoking status, Townsend deprivation index, LDL-cholesterol level, and blood glucose level) to further assess the possible role of other risk factors on the association. Finally, we conducted a sensitivity analysis and re-constructed a genetic risk score for AF, excluding genetic variants associated with any type of well-known cardiovascular risk factors or diseases (Additional file 3: Figure S2).

### Genetic analysis for the effect of blood pressure-lowering drug classes

BP-lowering drug effects can be predicted through variants in genes that encode receptors related to the mechanism of action. For example, ADRB1 is a gene that encodes the adrenergic receptor beta 1, present in cardiomyocytes and the heart conduction system, thus, modulating inotropy and chronotropy. Beta-blockers prevent activation of those receptors by adrenaline and noradrenaline and, hence, have a negative inotropic and chronotropic effect [26]. Therefore, genetic variants in the ADRB1 gene associated with SBP can be used as a proxy for exposure to beta-blockers and thus help predict the effect of that drug class on the risk of AF. We used the approach suggested by Gill et al. [27] to select genetic variants that mimic the effect of each BP-lowering drug class. Details of variant selection have been reported by Gill et al. [27]. Two-sample MR method through the inverse-variance weighted approach was used for the statistical analysis. We used the ratio of coefficients (Wald ratio) method as an alternative approach when only a single variant was available [28]. The same GWAS studies for SBP and AF were used for this stage of the analysis [7, 14].

## Results

### Elevated systolic blood pressure increases the risk of atrial fibrillation

**Fig. 2:**
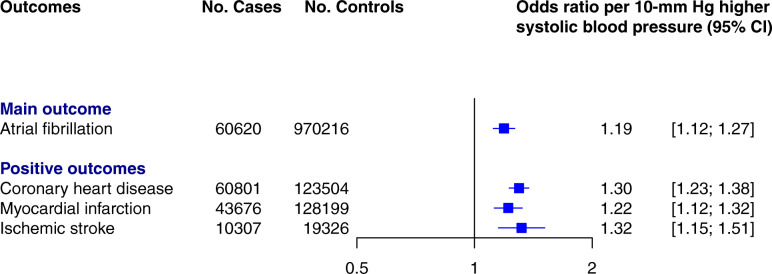
Two-sample Mendelian randomization estimates for the association between genetically predicted systolic blood pressure per 10-mmHg and atrial fibrillation as the main outcome, and coronary heart disease, myocardial infarction, and ischemic stroke as positive control outcomes. Solid squares represent point estimates and vertical lines 95% confidence intervals (CI). Cases and controls: number of cases and controls in genome-wide association studies. Odds ratio estimated using the inverse-variance weighted method

Using the inverse-variance weighted method, each 10-mmHg genetically predicted higher SBP was associated with a 19% higher risk of AF (odds ratio [OR] 1.19 [95% CI 1.12 to 1.27], *p* < 0.001) (Fig. 2). The strong associations between SBP and coronary heart disease (OR 1.30 [95% CI 1.23 to 1.38], *p* < 0.001), myocardial infarction (OR 1.22 [95% CI 1.12 to 1.32], *p* < 0.001), and ischemic stroke (OR 1.32 [95% CI 1.15 to 1.51], *p* < 0.001) suggest that the instrumental variable was valid. The MR regression slopes are shown in Additional file 3: Figures S4 and S5. Overall, estimated ORs based on different MR methods were similar, except the MR-Egger method that showed no effect and wide CIs (Additional file 3: Figure S6). However, after excluding outlier variants, the MR-Egger estimation was consistent with the main estimation and other sensitivity analyses (Additional file 3: Figure S6). There was evidence of directional pleiotropy based on MR-Egger intercept (beta 0.004; [standard error = 0.002] *p* = 0.02), which was considerably diluted after excluding outlier variants (beta = 0.001; [standard error = 0.001] *p* = 0.54). In the leave-one-out analysis, we found that no single genetic variant was strongly driving the overall effect of SBP on AF (Additional file 3: Figure S7). Sensitivity analysis using a series of sequentially restricted linkage disequilibrium threshold for clumping approved no material change in the main estimation (Additional file 3: Figure S8). The multivariable MR analysis showed similar findings before and after adjustment for BMI (Additional file 3: Figure S9).

### The stronger effect in participants with greater genetic susceptibility of atrial fibrillation

**Fig. 3:**
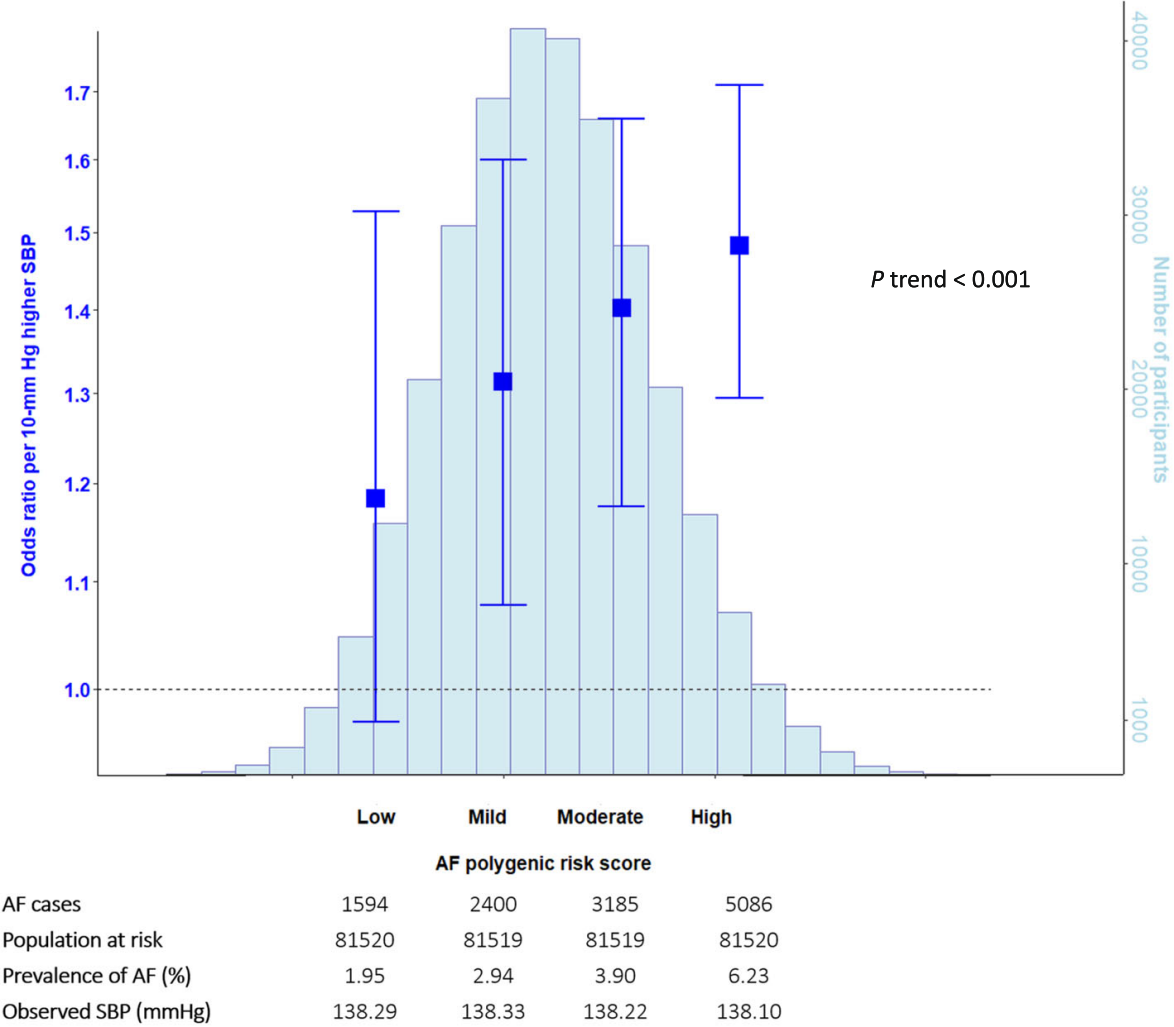
Stratified Mendelian randomization for the effect of genetically predicted higher systolic blood pressure and risk of atrial fibrillation by genetic susceptibility for atrial fibrillation. The analysis adjusted for age, sex, genotype measurement batch, genetic kinship to other participants, UK Biobank assessment center, and first ten genetic principal components (population stratification adjustment). AF, atrial fibrillation; SBP, systolic blood pressure

Additional file 2: Table S3 describes the characteristics of 329,237 UK Biobank participants with and without AF. We identified 12,391 cases of AF (prevalence 3.7% [95% CI 3.6 to 3.8%]) through diagnostic codes in the UK Biobank. The overall finding of this one-sample analysis was in keeping with the two-sample analysis (Additional file 3: Figures S6 and S10). Risk-stratified analyses further showed evidence for interaction in the relative effect of SBP on AF. We found no material difference in *measured* SBP values by AF genetic risk score (mean SBP, 138 mmHg in all subgroups including low, mild, moderate, and high genetic susceptibility for AF), but a linear gradient increase in causal association between SBP and AF risk was observable (Fig. 3). When stratified according to the AF genetic risk score, the causal effect of increasing SBP on AF risk was least in participants with low genetic risk (OR 1.20 [95% CI 0.95 to 1.53]), modest in those with mild or moderate genetic risk (OR for mild, 1.34 [95% CI 1.10 to 1.63], and OR for moderate, 1.43 [95% CI 1.20 to 1.69]), and strongest among those with high genetic risk (OR 1.51 [95% CI 1.32 to 1.74]) (Fig. 3).

#### Sensitivity analyses

The positive control findings further support the validity of the analyses, confirming the causal link between SBP and coronary heart disease, myocardial infarction and stroke (Additional file 3: Figure S10). Also, exclusion of all patients with the diagnosis of coronary heart disease, heart failure, and valvular heart disease in the UK biobank led to no material change in the one-sample MR overall estimation (Additional file 3: Figure S11). Finally, the rest of the sensitivity analyses showed that the analyses based on different case definitions, adjustment levels, and modified genetic risk score for AF were in line with the main results (Additional file 3: Figures S12 and S13, Additional file 2: Table S4).

### Prediction of BP-lowering drug effects

**Fig. 4:**
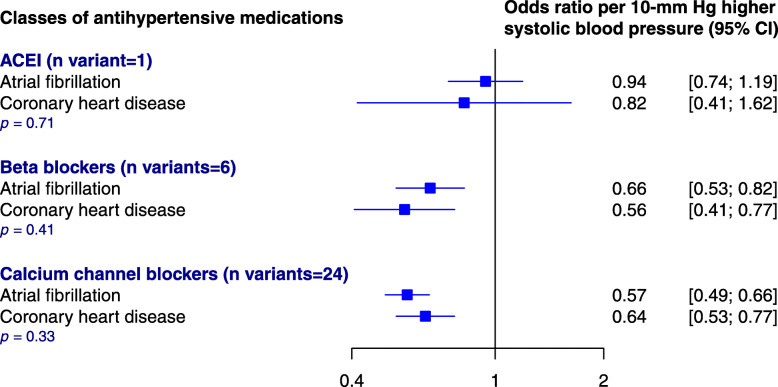
Association of genetically predicted systolic blood pressure reduction for each major class of antihypertensive medications on the risk of atrial fibrillation or coronary heart disease. CI, confidence interval; ACEI, angiotensin-converting enzyme inhibitors; n, number; *p*, *p* value for heterogeneity between two outcomes, calculated using the chi-square test. Odds ratio estimated using the inverse-variance weighted method

Candidate variants have been previously reported for angiotensin-converting enzyme inhibitors (ACEI) (one variant), beta-blockers (6 variants), and calcium channel blockers (24 variants), enabling investigation of effects by major drug classes (Additional file 2: Table S5) [27]. Each 10-mmHg decrement in SBP determined through genetic variants for ACEI, beta-blockers, and calcium channel blockers classes showed similar magnitude and direction of effects to those for coronary heart disease, as an established evidence-based target for preventive BP-lowering treatment (Fig. 4).

## Discussion

This study has shown that exposure to a higher SBP increases the risk of AF by 19% per each 10-mmHg higher SBP, but this average effect varied according to genetic susceptibility to AF. While there was no clear evidence of an increased risk in people with low genetic susceptibility, those with a high genetic predisposition have a 51% increase in AF risk for each 10-mmHg increment in SBP. Furthermore, by using genetic variants for three major antihypertensive drug classes as corollary evidence, we have shown a similar relative effect on AF prevention compared to their effects on coronary heart disease. Findings from this study complement those seen in previous research and may help explain some of the conflicting findings between epidemiological observational studies and clinical trials.

Previous MR study, with the main focus on the causal pathway from ischemic stroke to atrial fibrillation, reported a similar causal effect between SBP and AF [29]. The association we found is broadly consistent with a previous large-scale observational cohort study, which suggested a 10% increase in risk per 10-mmHg higher SBP [2]. The stronger association for the same magnitude of increase in SBP found in our study is likely indicative of lifelong risk exposure measured in MR studies as opposed to more limited follow-up duration in conventional prospective cohort studies. More importantly, our analyses support the causal nature of this association by mitigating reverse causality and confounding as potential alternative explanations. Moreover, we report the consistency of effect estimates for three major antihypertensive classes on AF and coronary heart disease, which provides further evidence for the modifiable nature of the risk of AF through the use of common antihypertensive drugs.

One of the major implications of our research is the finding that the same level of BP-lowering treatment might have quantitatively different relative (and absolute) effects in people with varying genetic predisposition for AF, despite having phenotypically similar SBP values. This could offer a strategy for the selection of a group of individuals in whom treatment might have no or little preventative effect and help target interventions on those with the highest genetic risk of development of AF. This is in line with the ambition of precision or stratified medicine [30], despite inherent limitations to its scope [31] and often lack of rigorous analytical techniques to support its claims [32]. In particular, in the context of common multi-cause cardiovascular diseases such as AF, evidence for the value of stratified treatment remains limited. A common approach by previous genetic studies has been to search for “treatment responders” through stratification of patients based on their genetic susceptibility for a particular drug target (i.e., exposure) with often disappointing results [33]. For instance, findings from the Heart Protection Study revealed that the risk reduction of major vascular outcomes during 5 years of statin therapy was not different across common genetic variants associated with lipid response [33].

An alternative strategy is to stratify individuals based on the risk of the outcome that is to be prevented. A commonly adopted approach here is to perform a subgroup analysis based on *clinical* risk scores. However, previous individual patient data (IPD) meta-analyses of trials that stratified participants based on baseline clinical risk of cardiovascular disease showed that the proportional risk reductions during 5 years of statin therapy or BP-lowering therapy to be largely similar across risk categories [34, 35]. The lack of treatment interaction by clinical risk in these studies might be due to the relatively narrow range of clinical risk of trial populations or the fact that increasing age, which is often a strong predictor of clinical risk, counteracts the expected higher relative effects in high-risk groups [36].

Genetic risk scores capture lifelong risks and overcome these issues, particularly when the predictive ability of conventional clinical risk scores is not as high as genetic risk scores, which seems to be the case in AF [37]. We are not aware of any prior study that focused on the effect of BP-lowering drugs and stratified patients by their genetic susceptibility for AF or other cardiovascular outcomes. Our study fills this gap in evidence and broadly follows the same principle as in an earlier IPD meta-analysis of four statin trials which stratified patients by their genetic risk for coronary heart disease and demonstrated a gradient in relative risk reductions across low to high genetic risk categories (13 to 48%) [38].

Although our study provides a strong foundation for the hypothesis that BP-lowering treatment could be more beneficial in patients with higher genetic risk of AF, RCTs with access to genetic information are necessary to confirm its findings and to compare perhaps the value of AF genetic risk scoring against other potential indicators of risk. However, the high resources required for such trials, particularly in the presence of compelling evidence for the efficacy of BP-lowering to reduce the risk of adverse cardiovascular events [39], may render conduct and design of trials with AF as their primary outcome unfeasible or unethical. To our knowledge, no RCT of antihypertensive treatment with AF as its primary outcome has been published or is currently ongoing. However, several major trials of antihypertensive drugs have reported incidence of AF as their secondary outcomes or through adverse event reporting. The largest tabular meta-analysis of these trials showed substantial heterogeneity in effects on risk of AF across trials, with a 25% risk reduction in patients with heart failure but no effect in patients without heart failure [6]. The same meta-analysis reported a graded effect by the trial-level rate of AF, with a risk ratio (RR) of 0.86 (95% CI 0.81 to 0.93) for trials in the highest event rate versus a RR of 0.98 (95% CI 0.88 to 1.09) for lowest event rate. This observation raised the hypothesis that the overall null findings in the non-heart failure trials might have been due to the inclusion of participants at low risk of AF. However, the study lacked data to estimate risk at the level of individuals and could not adequately take account of other important determinants of treatment effect such as the intensity of BP reduction or disease interaction, in particular for heart failure, where mechanisms of antihypertensive drugs might differ from hypertension [40]. Future analyses of individual-participant data from a consortium of large-scale BP-lowering trials could investigate this question in more detail [41]. Our findings could also inform future trial designs for an enriched selection of trial participants to test this hypothesis directly.

Our findings should be interpreted considering the limitations of this study. First, we used AF cases from linked hospital electronic health records, and a degree of misclassification could not be excluded. However, previous studies have revealed that the diagnostic validity of AF in electronic health records is above 85% compared with electrocardiographic assessment, and any misclassification is only expected to have diluted the observed associations [42]. Second, MR analysis assumes that there is no alternative causal pathway and the variants selected as an instrumental variable for SBP influence AF only through the exposure of interest (i.e., no pleiotropic effect). Although it is impossible to be certain that the variants used in this study do not have pleiotropic effects, we did not find any strong evidence in favor of the pleiotropic effect using extensive sensitivity analyses. Finally, our study was restricted to a population of European descent for the sake of genetic homogeneity. However, this limits the generalizability of the observed associations to other ethnicities with different genetic backgrounds.

## Conclusions

This study shows that the association between elevated BP and increased risk of AF is likely to be causal, with a more pronounced impact in individuals with high genetic susceptibility for AF. In the absence of clinical trials, this study provides another indication for BP-lowering treatment, in particular, among individuals at high genetic risk for AF. The concept of stratifying management based on an individual’s genetic susceptibility for AF seems to overcome some of the challenges of identification of treatment responders or simple clinical risk scores that have often failed to show a modifying effect on treatment effects. Investigations into the value of such outcome- versus exposure-based genetic susceptibility selection for other multi-cause outcomes would be of great relevance in “precision” or “stratified” medicine.

## Supplementary Information

**Additional file 1.** Details of various sensitivity analysis methods of two-sample Mendelian randomization. Details of inclusion and exclusion criteria for one-sample Mendelian randomization using UK biobank individual participant data. Details of genetic risk score development for atrial fibrillation. Details of statistical analysis for one-sample Mendelian randomization. Diagnosis codes for identification of outcomes in the UK Biobank cohort study.

**Additional file 2: Table S1.** Genetic variants were selected as an instrumental variable for systolic blood pressure. **Table S2.** Characteristics of variants used to develop genetic risk score to determine genetic susceptibility of atrial fibrillation for each person. **Table S3.** Summary of characteristics of 329,237 participants of white British ancestry included in the one-sample stratified Mendelian randomization. **Table S4.** Sensitivity analysis using a modified generic risk score for atrial fibrillation. **Table S5.** Characteristics of genetic variants used as proxies for classes of blood pressure-lowering drug effects.

**Additional file 3. Figure S1.** The genetic variant selection workflow for systolic blood pressure. **Figure S2.** The genetic variant selection workflow for atrial fibrillation. **Figure S3.** Distribution of atrial fibrillation genetic risk score created by 102 genetic variants on 329,237 participants of the UK Biobank with valid genetic data and full blood pressure measurement. **Figure S4.** Scatter plot of 254 genetic variants associated with systolic blood pressure and their effect on atrial fibrillation. **Figure S5.** Funnel plot of 254 variants, showing each variant causal estimate against instrument strength. **Figure S6.** The association between systolic blood pressure and risk of atrial fibrillation estimated by random-effect inverse variance weighted and applied various sensitivity analysis methods of two-sample Mendelian randomization. **Figure S7.** Leave-one-out plot to assess if a single genetic variant is driving the association between systolic blood pressure and atrial fibrillation. **Figure S8.** Sensitivity analysis for two-sample Mendelian randomization using a series of sequentially restricted linkage disequilibrium threshold for clumping. **Figure S9.** Multivariable Mendelian randomization results unadjusted and adjusted for body mass index to check the possibility of collider bias in association between systolic blood pressure and atrial fibrillation. **Figure S10.** One-sample Mendelian randomization for the association between systolic blood pressure per 10-mmHg and coronary heart disease and stroke as positive outcomes. **Figure S11.** One-sample Mendelian randomization for the association between systolic blood pressure per 10-mmHg and atrial fibrillation with the exclusion of all prevalent or incidence cases of coronary heart disease, heart failure and valvular heart disease. **Figure S12.** Sensitivity analysis for assessing the effect of case definition through ICD codes and self-report on the main estimation. **Figure S13.** Sensitivity analysis for assessing the effect of further adjustment for well-known cardiovascular risk factors on the main estimation.

## Data Availability

Access to summary statistics for atrial fibrillation is available from http://csg.sph.umich.edu/willer/public/afib2018/ [7] and for ICBP consortium from https://www.ncbi.nlm.nih.gov/projects/gap/cgi-bin/study.cgi?study_id=phs000585.v2.p1 [14, 43, 44]. All bona fide researchers can apply to use the UK Biobank dataset for health-related research. A guide to access is also provided at http://www.ukbiobank.ac.uk/register-apply/. The detailed results supporting the conclusions of this article are included within the article and its supplementary materials.
