# Supplementary material for: Genetic susceptibility, elevated blood pressure, and risk of atrial fibrillation: a Mendelian randomization study
Source: Genome Med. 2021 Mar 4;13:38. doi: 10.1186/s13073-021-00849-3 (PMC7934395; doi:10.1186/s13073-021-00849-3)
Supplement: Supplementary file 1 — Additional file 1. Details of various sensitivity analysis methods of two-sample Mendelian randomization. Details of inclusion and exclusion criteria for one-sample Mendelian randomization using UK biobank individual participant data. Details of genetic risk score development for atrial fibrillation. Details of statistical analysis for one-sample Mendelian randomization. Diagnosis codes for identification of outcomes in the UK Biobank cohort study. [file 13073_2021_849_MOESM1_ESM.docx]

## **Supplementary methods**

## Details of various sensitivity analysis methods of two-sample Mendelian randomization

The inverse-variance weighted method assumes that either all the instruments are valid or any horizontal pleiotropy is balanced.[1] We provided an estimation using the weighted median method [2], which is consistent if at least 50% of the weight comes from valid instrumental variables.[3] The Mendelian randomization pleiotropy residual sum and outlier (MR-PRESSO) method was used to test and, if needed, correct for any possible horizontal pleiotropic outliers in the analysis.[4] Robust Adjusted Profile Score (RAPS) estimator is robust to systematic and idiosyncratic pleiotropy and is recommended for complex traits and diseases.[5] Mendelian randomization analysis using mixture models (MRMix) method provides unbiased estimation in the presence of a large number of invalid genetic instruments. [6] The MR-Egger regression method was used to account for potential pleiotropy.[7] MR-Egger is prone to low statistical power, and therefore as an additional sensitivity analysis, we applied the mode-based estimate (MBE). [1] The statistical power of the MBE method has been reported to have lower power than the inverse-variance weighting method and weighted median methods but larger than MR-Egger.[1] Also, MR-Egger is susceptible to outlier genetic variants. [8] To address this issue, in a sensitivity analysis, first, we calculated Cook’s distance measure [8, 9] to detect the outlier variants and then reran the MR-Egger analysis after removing the outlier variants.

## Details of inclusion and exclusion criteria for one-sample Mendelian randomization using UK biobank individual participant data.

Genotype data were imputed with IMPUTE4 using the Haplotype Reference Consortium (HRC) and the UK10K + 1000 Genomes panel [10] to identify ~ 96 million variants for 487,381 participants. “From 419,056 white descent (self-reported Irish, British or other white backgrounds) with valid genetic and blood pressure measurements, we excluded non-Caucasian participants (n=46,071), missingness rate (variant call rate) higher than 1% (n=42,773), mismatch between reported and genetic sex (n=257), participants with sex chromosome aneuploidy (n=311), and UK biobank recommended genomic analysis exclusions (n=407)”.[11] The weighted genetic risk score (GRS) for elevated systolic blood pressure was used as an instrumental variable. It was developed using 254 variants (**Figure S1**) from the GWAS by Evangelou et al. [12] and weighted by beta coefficients derived from International Consortium for Blood Pressure (ICBP) GWAS. [13]

## Details of genetic risk score development for atrial fibrillation.

“First, each variant was recoded additively (0, 1, and 2) according to the number of alleles that increase the log odds of atrial fibrillation. Then, each variant was weighed according to the logistic regression coefficient obtained from the GWAS meta-analysis to give more weight to variants with stronger effects. A weighted genetic risk score was constructed using the following formula: (β_1_×SNP_1_(+)β_2_×SNP_2_(+⋯(β_n_×SNP_n_), where βi was the regression coefficient associated with SNPi and obtained from the GWAS study.” [11] There was no overlap between these variants and variants selected for systolic blood pressure as instrument. Additionally, we conducted a sensitivity analysis using a modified genetic risk score for atrial fibrillation. First, the individual association of each variant with other risk factors or cardiovascular diseases has been checked using the NHGRI-EBI Catalog of published genome-wide association studies (https://www.ebi.ac.uk/gwas/home). We then conducted a sensitivity analysis and re-constructed a genetic risk score, excluding genetic variants associated with any type of well-known cardiovascular risk factors or diseases.

## Details of statistical analysis for one-sample Mendelian randomization

The analysis was performed using an adjusted two-stage predictor substitution method that used the elevated systolic blood pressure genetic risk score as instrumental variable.[14] In this analysis, we regressed measured systolic blood pressure on the genetic risk score as instrumental variable using a linear regression model. The predicted probability and residuals were then derived from this model (the predicted value is a proxy for an unconfounded estimate of systolic blood pressure). Binary outcomes of atrial fibrillation were then regressed on the predicted probability in a multi-adjusted binary logistic regression model with robust standard errors. The analysis was adjusted for age, sex, UK Biobank assessment center, genotype measurement batch, genetic kinship to other participants and first ten genetic principal components.

## Diagnosis codes for identification of outcomes in the UK Biobank cohort study

**Atrial Fibrillation**

Description: diagnoses - main and secondary ICD10

I48.0 Paroxysmal atrial fibrillation

I48.1 Persistent atrial fibrillation

I48.2 Chronic atrial fibrillation

I48.3 Typical atrial flutter

I48.4 Atypical atrial flutter

I48.9 Atrial fibrillation and atrial flutter, unspecified

Description: diagnoses – main and secondary ICD9

4273 Atrial fibrillation and flutter

Description: Non-cancer illness code, self-reported

1471 Atrial fibrillation

1483 Atrial flutter

**Coronary heart disease**

Description: diagnoses – main and secondary ICD10

I25.0 Atherosclerotic cardiovascular disease, so described

I25.1 Atherosclerotic heart disease

I25.3 Aneurysm of heart

I25.4 Coronary artery aneurysm

I25.5 Ischaemic cardiomyopathy

I25.6 Silent myocardial ischaemia

I25.8 Other forms of chronic ischaemic heart disease

I25.9 Chronic ischaemic heart disease, unspecified

Description: diagnoses – main and secondary ICD9

4140 Coronary atherosclerosis

**Myocardial Infarction**

(**Derived from UK Biobank Outcome Adjudication Group)**

Description: Non-cancer illness code, self-reported

Field 20002 Code 1075 Heart attack/myocardial infarction

Description: diagnoses - main and secondary ICD10

I21 Acute myocardial infarction

I21.0 Acute transmural myocardial infarction of anterior wall

I21.1 Acute transmural myocardial infarction of inferior wall

I21.2 Acute transmural myocardial infarction of other sites

I21.3 Acute transmural myocardial infarction of unspecified site

I21.4 Acute subendocardial myocardial infarction

I21.9 Acute myocardial infarction, unspecified

I22 Subsequent myocardial infarction

I22.0 Subsequent myocardial infarction of anterior wall

I22.1 Subsequent myocardial infarction of inferior wall

I22.8 Subsequent myocardial infarction of other sites

I22.9 Subsequent myocardial infarction of unspecified site

I23 Certain current complications following acute myocardial infarction

I23.0 Haemopericardium as current complication following acute myocardial infarction

I23.1 Atrial septal defect as current complication following acute myocardial infarction

I23.2 Ventricular septal defect as current complication following acute myocardial infarction

I23.3 Rupture of cardiac wall without haemopericardium as current complication following acute myocardial infarction

I23.4 Rupture of chordae tendineae as current complication following acute myocardial infarction

I23.5 Rupture of papillary muscle as current complication following acute myocardial infarction

I23.6 Thrombosis of atrium, auricular appendage, and ventricle as current complications following acute myocardial infarction

I23.8 Other current complications following acute myocardial infarction

I24.1 Dressler syndrome

I25.2 Old myocardial infarction

Description: diagnoses – main and secondary ICD9

410 Acute myocardial infarction

410.0 Acute myocardial infarction of anterolateral wall

410.1 Acute myocardial infarction of other anterior wall

410.2 Acute myocardial infarction of inferolateral wall

410.3 Acute myocardial infarction of inferoposterior wall

410.4 Acute myocardial infarction of other inferior wall

410.5 Acute myocardial infarction of other lateral wall

410.6 True posterior wall infarction

410.7 Subendocardial infarction

410.8 Acute myocardial infarction of other specified sites

410.9 Acute myocardial infarction of unspecified site

411 Other acute and subacute forms of ischaemic heart disease

411.0 Postmyocardial infarction syndrome

411.1 Intermediate coronary syndrome

411.8 Other

**Heart Failure**

Description: Non-cancer illness code, self-reported

1076 Heart failure/pulmonary odema

Description: diagnoses – main and secondary ICD9

4280 Congestive heart failure

4281 Left heart failure

Description: diagnoses – main and secondary ICD10

I50.0 Congestive heart failure

I50.1 Left ventricular failure

I50.9 Heart failure, unspecified

**Valvular Heart Disease**

Description: diagnoses – main and secondary ICD10

I06.2 Rheumatic aortic stenosis with insufficiency

I06.0 Rheumatic aortic stenosis

I35.2 Aortic (valve) stenosis with insufficiency

I35.0 Aortic (valve) stenosis

I06.1 Rheumatic aortic insufficiency

I35.1 Aortic valve insufficiency

I05.0 Mitral stenosis

I05.2 Mitral stenosis with insufficiency

I34.2 Nonrheumatic mitral (valve) stenosis

I05.1 Rheumatic mitral insufficiency

I34.0 Mitral (valve) insufficiency

Description: Non-cancer illness code, self-reported

1490 Aortic stenosis

1587 Aortic regurgitation / incompetence

1489 Mitral stenosis

1585 mitral regurgitation / incompetence

**References**

1. Hartwig FP, Smith GD, Bowden J. Robust inference in summary data Mendelian randomization via the zero modal pleiotropy assumption. International Journal of Epidemiology. 2017;46:1985–98.

2. Hemani G, Bowden J, Davey Smith G. Evaluating the potential role of pleiotropy in Mendelian randomization studies. Human molecular genetics. 2018;27:R195–208.

3. Bowden J, Davey Smith G, Haycock PC, Burgess S. Consistent Estimation in Mendelian Randomization with Some Invalid Instruments Using a Weighted Median Estimator. Genetic epidemiology. 2016;40:304–14. doi:10.1002/gepi.21965.

4. Verbanck M, Chen C-Y, Neale B, Do R. Detection of widespread horizontal pleiotropy in causal relationships inferred from Mendelian randomization between complex traits and diseases. Nature Genetics. 2018;50:693–8. doi:10.1038/s41588-018-0099-7.

5. Zhao Q, Wang J, Hemani G, Bowden J, Small DS. Statistical inference in two-sample summary-data Mendelian randomization using robust adjusted profile score. Annals of Statistics. 2018;48:1742–69. http://arxiv.org/abs/1801.09652. Accessed 19 Oct 2020.

6. Qi G, Chatterjee N. Mendelian randomization analysis using mixture models for robust and efficient estimation of causal effects. Nature Communications. 2019;10:1–10. doi:10.1038/s41467-019-09432-2.

7. Bowden J, Davey Smith G, Burgess S. Mendelian randomization with invalid instruments: effect estimation and bias detection through Egger regression. International Journal of Epidemiology. 2015;44:512–25. doi:10.1093/ije/dyv080.

8. Burgess S, Thompson SG. Interpreting findings from Mendelian randomization using the MR-Egger method. European Journal of Epidemiology. 2017;32:377–89. doi:10.1007/s10654-017-0255-x.

9. Cook RD. Detection of Influential Observation in Linear Regression. Technometrics. 1977;19:15.

10. Bycroft C, Freeman C, Petkova D, Band G, Elliott LT, Sharp K, et al. The UK Biobank resource with deep phenotyping and genomic data. Nature. 2018;562:203–9. doi:10.1038/s41586-018-0579-z.

11. Nazarzadeh M, Pinho-Gomes AC, Smith Byrne K, Canoy D, Raimondi F, Ayala Solares JR, et al. Systolic Blood Pressure and Risk of Valvular Heart Disease: A Mendelian Randomization Study. JAMA Cardiology. 2019;4:788–95. doi:10.1001/jamacardio.2019.2202.

12. Evangelou E, Warren HR, Mosen-Ansorena D, Mifsud B, Pazoki R, Gao H, et al. Genetic analysis of over 1 million people identifies 535 new loci associated with blood pressure traits. Nature Genetics. 2018. doi:10.1101/198234.

13. Ehret GB, Munroe PB, Rice KM, Bochud M, Johnson AD, Chasman DI, et al. Genetic variants in novel pathways influence blood pressure and cardiovascular disease risk. Nature. 2011;478:103–9. doi:10.1038/nature10405.

14. Burgess S, Thompson SG. Mendelian randomization: methods for using genetic variants in causal estimation. Taylor & Francis Group; 2015.
