# Supplementary material for: Genetic susceptibility, elevated blood pressure, and risk of atrial fibrillation: a Mendelian randomization study
Source: Genome Med. 2021 Mar 4;13:38. doi: 10.1186/s13073-021-00849-3 (PMC7934395; doi:10.1186/s13073-021-00849-3)
Supplement: Supplementary file 2 — Additional file 2: Table S1. Genetic variants were selected as an instrumental variable for systolic blood pressure. Table S2. Characteristics of variants used to develop genetic risk score to determine genetic susceptibility of atrial fibrillation for each person. Table S3. Summary of characteristics of 329,237 participants of white British ancestry included in the one-sample stratified Mendelian randomization. Table S4. Sensitivity analysis using a modified generic risk score for atrial fibrillation. Table S5. Characteristics of genetic variants used as proxies for classes of blood pressure-lowering drug effects. [file 13073_2021_849_MOESM2_ESM.docx]

| Table S1: Genetic variants were selected as an instrumental variable for systolic blood pressure. | | | | | | | | | |
| --- | --- | --- | --- | --- | --- | --- | --- | --- | --- |
| SNP * | Chromosome | Position (GRCh37) | Allele1 | Allele2 | Freq1† | Effect † | Standard error † | P-value† | Source * |
| rs3737801 | 1 | 27960832 | c | g | 0.9142 | 0.4246 | 0.0954 | 8.67E-06 | Novel:one-stage design |
| rs11210029 | 1 | 41865293 | a | g | 0.625 | -0.1608 | 0.0476 | 0.000728 | Novel:one-stage design |
| rs11579440 | 1 | 49052423 | t | c | 0.8468 | 0.2794 | 0.0653 | 1.86E-05 | Novel:one-stage design |
| rs10923038 | 1 | 88651771 | a | c | 0.6166 | 0.1279 | 0.0481 | 0.00781 | Novel:one-stage design |
| rs76719272 | 1 | 156129796 | t | c | 0.1309 | -0.2747 | 0.0727 | 0.00016 | Novel:two-stage design |
| rs1043069 | 1 | 180859368 | t | g | 0.6225 | 0.2696 | 0.0478 | 1.72E-08 | Novel:two-stage design |
| rs4651224 | 1 | 184585182 | t | c | 0.4518 | 0.144 | 0.047 | 0.002185 | Novel:two-stage design |
| rs12042924 | 1 | 197297417 | t | c | 0.5202 | -0.1372 | 0.0465 | 0.003202 | Novel:two-stage design |
| rs33996239 | 1 | 203109801 | t | c | 0.0577 | -0.427 | 0.1058 | 5.43E-05 | Novel:two-stage design |
| rs7555285 | 1 | 209970355 | c | g | 0.7951 | 0.173 | 0.0565 | 0.002212 | Novel:two-stage design |
| rs260508 | 1 | 2187085 | t | g | 0.6167 | 0.1696 | 0.0477 | 0.000377 | Novel:two-stage design |
| rs2807337 | 1 | 22577371 | t | c | 0.3721 | 0.1938 | 0.0478 | 5.02E-05 | Novel:two-stage design |
| rs4926499 | 1 | 249155909 | c | g | 0.8262 | 0.2922 | 0.0752 | 0.000102 | Novel:two-stage design |
| rs79598313 | 1 | 27284913 | t | c | 0.0275 | 0.5126 | 0.1518 | 0.00073 | Novel:two-stage design |
| rs839755 | 1 | 43856410 | a | c | 0.6224 | -0.1877 | 0.047 | 6.55E-05 | Novel:two-stage design |
| rs7514579 | 1 | 94051350 | a | c | 0.7765 | 0.3027 | 0.0559 | 6.16E-08 | Novel:two-stage design |
| rs17396055 | 1 | 94730954 | a | g | 0.3317 | -0.1525 | 0.049 | 0.001874 | Novel:two-stage design |
| rs880315 | 1 | 10796866 | t | c | 0.652 | -0.5218 | 0.0499 | 1.33E-25 | Previously reported |
| rs4846049 | 1 | 11850365 | t | g | 0.3264 | -0.4146 | 0.0489 | 2.44E-17 | Previously reported |
| rs17367504 | 1 | 11862778 | a | g | 0.8444 | 0.7774 | 0.0639 | 4.81E-34 | Previously reported |
| rs5068 | 1 | 11905974 | a | g | 0.9387 | 1.0914 | 0.0989 | 2.53E-28 | Previously reported |
| rs3820068 | 1 | 15798197 | a | g | 0.7977 | 0.3361 | 0.0596 | 1.69E-08 | Previously reported |
| rs7515635 | 1 | 42408070 | t | c | 0.4684 | 0.2382 | 0.0463 | 2.70E-07 | Previously reported |
| rs10922502 | 1 | 89360158 | a | g | 0.6407 | -0.2283 | 0.0483 | 2.32E-06 | Previously reported |
| rs55732192 | 2 | 162278233 | t | g | 0.0962 | -0.2807 | 0.0798 | 0.000433 | Novel:one-stage design |
| rs6712203 | 2 | 165557318 | t | c | 0.3779 | -0.1943 | 0.0477 | 4.57E-05 | Novel:one-stage design |
| rs11694601 | 2 | 174949358 | a | g | 0.5927 | -0.1422 | 0.047 | 0.00249 | Novel:one-stage design |
| rs1837164 | 2 | 178716601 | a | t | 0.3753 | 0.186 | 0.0472 | 8.27E-05 | Novel:one-stage design |
| rs296797 | 2 | 201102905 | t | c | 0.4142 | 0.2067 | 0.0467 | 9.68E-06 | Novel:one-stage design |
| rs1047891 | 2 | 211540507 | a | c | 0.3243 | -0.1647 | 0.0511 | 0.00127 | Novel:one-stage design |
| rs10189186 | 2 | 53025757 | a | g | 0.5357 | 0.1752 | 0.0459 | 0.000135 | Novel:one-stage design |
| rs28377357 | 2 | 112769721 | a | g | 0.3 | -0.1588 | 0.0502 | 0.001572 | Novel:two-stage design |
| rs6723509 | 2 | 122000745 | t | c | 0.8617 | 0.2553 | 0.0677 | 0.000162 | Novel:two-stage design |
| rs72844590 | 2 | 138421227 | t | g | 0.1441 | 0.0856 | 0.0692 | 0.2158 | Novel:two-stage design |
| rs79523138 | 2 | 161368213 | a | g | 0.8849 | -0.3083 | 0.0749 | 3.83E-05 | Novel:two-stage design |
| rs6739913 | 2 | 185033065 | a | g | 0.7095 | -0.1523 | 0.0506 | 0.002638 | Novel:two-stage design |
| rs28558491 | 2 | 187816321 | t | c | 0.7362 | -0.1935 | 0.0531 | 0.000265 | Novel:two-stage design |
| rs67720684 | 2 | 18975439 | a | c | 0.2295 | 0.0834 | 0.0546 | 0.1269 | Novel:two-stage design |
| rs12694277 | 2 | 213188795 | t | c | 0.2914 | -0.219 | 0.051 | 1.77E-05 | Novel:two-stage design |
| rs1044822 | 2 | 230629138 | t | c | 0.142 | -0.2655 | 0.0657 | 5.38E-05 | Novel:two-stage design |
| rs139354822 | 2 | 242344695 | t | c | 0.9675 | 0.4794 | 0.1554 | 0.002042 | Novel:two-stage design |
| rs35590893 | 2 | 43716933 | a | g | 0.2716 | -0.1215 | 0.0515 | 0.01822 | Novel:two-stage design |
| rs6545155 | 2 | 50429861 | t | c | 0.7852 | 0.2182 | 0.0559 | 9.56E-05 | Novel:two-stage design |
| rs2920899 | 2 | 55279681 | t | g | 0.7851 | 0.1653 | 0.0573 | 0.003886 | Novel:two-stage design |
| rs72816333 | 2 | 60096560 | a | t | 0.8277 | 0.254 | 0.0606 | 2.79E-05 | Novel:two-stage design |
| rs2300481 | 2 | 66782467 | t | c | 0.3886 | 0.2043 | 0.0472 | 1.50E-05 | Novel:two-stage design |
| rs1446468 | 2 | 164963486 | t | c | 0.4512 | -0.487 | 0.0468 | 2.26E-25 | Previously reported |
| rs6712094 | 2 | 165043460 | a | g | 0.7296 | 0.42 | 0.0525 | 1.17E-15 | Previously reported |
| rs6749447 | 2 | 169041386 | t | g | 0.7323 | -0.067 | 0.0523 | 0.2008 | Previously reported |
| rs6434404 | 2 | 191494411 | a | g | 0.3247 | 0.2228 | 0.0498 | 7.53E-06 | Previously reported |
| rs1344653 | 2 | 19730845 | a | g | 0.4961 | -0.1568 | 0.0456 | 0.00058 | Previously reported |
| rs55780018 | 2 | 208526140 | t | c | 0.548 | -0.3278 | 0.0488 | 1.85E-11 | Previously reported |
| rs2972146 | 2 | 227100698 | t | g | 0.6362 | 0.2486 | 0.0476 | 1.76E-07 | Previously reported |
| rs55701159 | 2 | 25139596 | t | g | 0.887 | 0.2999 | 0.0742 | 5.27E-05 | Previously reported |
| rs1275988 | 2 | 26914364 | t | c | 0.6055 | -0.5157 | 0.0466 | 1.83E-28 | Previously reported |
| rs9678851 | 2 | 27887034 | a | c | 0.559 | -0.1135 | 0.0474 | 0.01662 | Previously reported |
| rs7562 | 2 | 28635740 | t | c | 0.5297 | 0.1555 | 0.047 | 0.00093 | Previously reported |
| rs13420463 | 2 | 37517566 | a | g | 0.7775 | 0.2751 | 0.0555 | 7.19E-07 | Previously reported |
| rs262986 | 3 | 183435713 | a | g | 0.4712 | -0.2288 | 0.0468 | 1.01E-06 | Novel:one-stage design |
| rs1882289 | 3 | 114461208 | a | g | 0.8814 | -0.2919 | 0.0708 | 3.78E-05 | Novel:two-stage design |
| rs9875380 | 3 | 132780356 | t | c | 0.4619 | -0.2472 | 0.0457 | 6.21E-08 | Novel:two-stage design |
| rs863930 | 3 | 135949737 | t | g | 0.4671 | -0.191 | 0.046 | 3.28E-05 | Novel:two-stage design |
| rs78151625 | 3 | 158316726 | t | c | 0.831 | -0.222 | 0.0618 | 0.000329 | Novel:two-stage design |
| rs189267552 | 3 | 20073193 | a | t | 0.0141 | -0.7415 | 0.2166 | 0.000618 | Novel:two-stage design |
| rs12638085 | 3 | 30405936 | a | t | 0.3525 | 0.2514 | 0.0486 | 2.30E-07 | Novel:two-stage design |
| rs6788984 | 3 | 41107173 | a | g | 0.858 | 0.3015 | 0.066 | 4.92E-06 | Novel:two-stage design |
| rs6774721 | 3 | 49381898 | a | g | 0.1465 | -0.2171 | 0.0689 | 0.001627 | Novel:two-stage design |
| rs9857362 | 3 | 74710462 | a | c | 0.5249 | 0.1736 | 0.0473 | 0.000241 | Novel:two-stage design |
| rs347591 | 3 | 11290122 | t | g | 0.6625 | 0.2842 | 0.0489 | 6.31E-09 | Previously reported |
| rs11128722 | 3 | 14958126 | a | g | 0.5628 | -0.2518 | 0.047 | 8.53E-08 | Previously reported |
| rs143112823 | 3 | 154707967 | a | g | 0.076 | -0.4019 | 0.0949 | 2.29E-05 | Previously reported |
| rs3097937 | 4 | 124794644 | a | t | 0.8075 | 0.2388 | 0.0587 | 4.77E-05 | Novel:one-stage design |
| rs6823767 | 4 | 151295085 | t | c | 0.7227 | -0.1566 | 0.0528 | 0.003027 | Novel:one-stage design |
| rs7439567 | 4 | 138464842 | t | c | 0.4157 | 0.245 | 0.0474 | 2.39E-07 | Novel:two-stage design |
| rs17035181 | 4 | 157678511 | t | g | 0.8549 | 0.2169 | 0.0653 | 0.000898 | Novel:two-stage design |
| rs2610990 | 4 | 18008232 | a | g | 0.2693 | -0.2325 | 0.0523 | 8.86E-06 | Novel:two-stage design |
| rs231708 | 4 | 2694773 | c | g | 0.6983 | -0.2643 | 0.0499 | 1.19E-07 | Novel:two-stage design |
| rs12511987 | 4 | 46595623 | t | g | 0.8224 | -0.2456 | 0.0614 | 6.26E-05 | Novel:two-stage design |
| rs1347345 | 4 | 95938386 | a | g | 0.6206 | -0.1645 | 0.0478 | 0.000583 | Novel:two-stage design |
| rs13112725 | 4 | 106911742 | c | g | 0.7682 | 0.397 | 0.0557 | 1.01E-12 | Previously reported |
| rs2291435 | 4 | 38387395 | t | c | 0.5248 | -0.2419 | 0.0463 | 1.74E-07 | Previously reported |
| rs2014912 | 4 | 86715670 | t | c | 0.1515 | 0.5122 | 0.0644 | 1.80E-15 | Previously reported |
| rs1650911 | 5 | 141740620 | c | g | 0.7619 | 0.2465 | 0.0584 | 2.42E-05 | Novel:one-stage design |
| rs12153395 | 5 | 179411477 | a | g | 0.1133 | -0.2602 | 0.0764 | 0.000661 | Novel:one-stage design |
| rs4957026 | 5 | 361148 | a | g | 0.3503 | 0.2214 | 0.0497 | 8.29E-06 | Novel:one-stage design |
| rs6875372 | 5 | 64079015 | a | t | 0.5154 | 0.2228 | 0.0459 | 1.18E-06 | Novel:one-stage design |
| rs1871190 | 5 | 97953719 | t | g | 0.3472 | 0.1658 | 0.0495 | 0.000805 | Novel:one-stage design |
| rs62373688 | 5 | 127352807 | a | t | 0.1259 | 0.3593 | 0.0714 | 4.76E-07 | Novel:two-stage design |
| rs10069690 | 5 | 1279790 | t | c | 0.2583 | 0.3827 | 0.0627 | 1.03E-09 | Novel:two-stage design |
| rs702395 | 5 | 140086677 | t | c | 0.4369 | 0.2367 | 0.0468 | 4.31E-07 | Novel:two-stage design |
| rs74774746 | 5 | 33411769 | c | g | 0.2639 | -0.1177 | 0.0541 | 0.02953 | Novel:two-stage design |
| rs13179413 | 5 | 55868097 | t | c | 0.2775 | 0.1383 | 0.0544 | 0.01098 | Novel:two-stage design |
| rs3121685 | 5 | 65662133 | t | c | 0.4815 | -0.2015 | 0.046 | 1.17E-05 | Novel:two-stage design |
| rs246973 | 5 | 68007803 | t | c | 0.2833 | 0.1984 | 0.0509 | 9.60E-05 | Novel:two-stage design |
| rs709668 | 5 | 96174186 | a | g | 0.1965 | -0.2755 | 0.0576 | 1.72E-06 | Novel:two-stage design |
| rs10077885 | 5 | 114390121 | a | c | 0.498 | -0.2465 | 0.0484 | 3.54E-07 | Previously reported |
| rs1008058 | 5 | 122435627 | a | g | 0.1183 | 0.3142 | 0.0766 | 4.12E-05 | Previously reported |
| rs13359291 | 5 | 122476457 | a | g | 0.1654 | 0.4005 | 0.062 | 1.06E-10 | Previously reported |
| rs6595838 | 5 | 127868199 | a | g | 0.2891 | 0.2361 | 0.0507 | 3.14E-06 | Previously reported |
| rs11953630 | 5 | 157845402 | t | c | 0.3694 | -0.4463 | 0.0501 | 5.15E-19 | Previously reported |
| rs1421811 | 5 | 32714270 | c | g | 0.6116 | 0.4743 | 0.0477 | 2.46E-23 | Previously reported |
| rs1173771 | 5 | 32815028 | a | g | 0.3976 | -0.5227 | 0.0468 | 6.04E-29 | Previously reported |
| rs10059921 | 5 | 87514515 | t | g | 0.0846 | -0.3732 | 0.0919 | 4.89E-05 | Previously reported |
| rs7765526 | 6 | 147713764 | a | g | 0.4682 | 0.2317 | 0.047 | 8.11E-07 | Novel:one-stage design |
| rs9449350 | 6 | 82281417 | t | c | 0.673 | -0.2333 | 0.0488 | 1.72E-06 | Novel:one-stage design |
| rs9401090 | 6 | 119113317 | t | c | 0.7538 | 0.2512 | 0.054 | 3.32E-06 | Novel:two-stage design |
| rs10782230 | 6 | 126228512 | a | g | 0.4907 | 0.2787 | 0.0459 | 1.27E-09 | Novel:two-stage design |
| rs9885632 | 6 | 131311909 | t | c | 0.7338 | 0.245 | 0.052 | 2.42E-06 | Novel:two-stage design |
| rs7763294 | 6 | 140383733 | t | g | 0.3169 | -0.2059 | 0.0493 | 2.95E-05 | Novel:two-stage design |
| rs2745599 | 6 | 1613686 | a | g | 0.5476 | 0.2128 | 0.0513 | 3.30E-05 | Novel:two-stage design |
| rs9368222 | 6 | 20686996 | a | c | 0.2767 | 0.1639 | 0.0511 | 0.001338 | Novel:two-stage design |
| rs6911827 | 6 | 22130601 | t | c | 0.4623 | 0.152 | 0.0473 | 0.001295 | Previously reported |
| rs2270860 | 6 | 43270151 | t | c | 0.3092 | 0.2966 | 0.05 | 3.09E-09 | Previously reported |
| rs10948071 | 6 | 43280713 | t | c | 0.5993 | -0.2074 | 0.0465 | 8.13E-06 | Previously reported |
| rs1563788 | 6 | 43308363 | t | c | 0.2937 | 0.3062 | 0.0501 | 9.79E-10 | Previously reported |
| rs78648104 | 6 | 50683009 | t | c | 0.8985 | -0.3571 | 0.083 | 1.69E-05 | Previously reported |
| rs35410524 | 6 | 96885405 | t | c | 0.1917 | 0.2999 | 0.0588 | 3.38E-07 | Previously reported |
| rs1870735 | 7 | 155744303 | c | g | 0.4548 | 0.2137 | 0.0486 | 1.08E-05 | Novel:one-stage design |
| rs12979 | 7 | 24738164 | c | g | 0.8745 | 0.2241 | 0.0693 | 0.001227 | Novel:one-stage design |
| rs34072724 | 7 | 130432469 | a | g | 0.4828 | -0.1967 | 0.0465 | 2.37E-05 | Novel:two-stage design |
| rs12703989 | 7 | 140238048 | a | g | 0.494 | 0.1026 | 0.0474 | 0.03035 | Novel:two-stage design |
| rs11771693 | 7 | 150050111 | a | g | 0.6743 | 0.169 | 0.0502 | 0.000757 | Novel:two-stage design |
| rs10274928 | 7 | 28142088 | a | g | 0.4932 | 0.1644 | 0.0475 | 0.000538 | Novel:two-stage design |
| rs10233127 | 7 | 30933453 | a | t | 0.1087 | 0.2638 | 0.0805 | 0.001051 | Novel:two-stage design |
| rs6593297 | 7 | 56122058 | a | t | 0.3178 | 0.0982 | 0.0523 | 0.06052 | Novel:two-stage design |
| rs6963105 | 7 | 75097488 | a | g | 0.4432 | -0.2035 | 0.0531 | 0.000127 | Novel:two-stage design |
| rs848445 | 7 | 77572461 | t | c | 0.2821 | -0.2067 | 0.0528 | 9.04E-05 | Novel:two-stage design |
| rs17477177 | 7 | 106411858 | t | c | 0.7906 | -0.5642 | 0.0564 | 1.60E-23 | Previously reported |
| rs4728142 | 7 | 128573967 | a | g | 0.4383 | -0.2155 | 0.0467 | 3.91E-06 | Previously reported |
| rs13238550 | 7 | 131059056 | a | g | 0.3909 | 0.1695 | 0.0472 | 0.000329 | Previously reported |
| rs10224002 | 7 | 151415041 | a | g | 0.7186 | -0.2375 | 0.0525 | 5.99E-06 | Previously reported |
| rs6969780 | 7 | 27159136 | c | g | 0.0961 | 0.3697 | 0.0793 | 3.12E-06 | Previously reported |
| rs142449193 | 8 | 102750597 | t | c | 0.0491 | -0.4354 | 0.112 | 0.000102 | Novel:one-stage design |
| rs4875958 | 8 | 1721090 | a | g | 0.7099 | 0.2209 | 0.0515 | 1.83E-05 | Novel:one-stage design |
| rs2979470 | 8 | 30288272 | t | c | 0.4873 | 0.2114 | 0.046 | 4.25E-06 | Novel:one-stage design |
| rs2354862 | 8 | 64501744 | a | c | 0.6441 | 0.2139 | 0.0485 | 1.03E-05 | Novel:one-stage design |
| rs13253358 | 8 | 68920135 | t | c | 0.297 | 0.1945 | 0.0504 | 0.000113 | Novel:one-stage design |
| rs61040371 | 8 | 8503700 | t | c | 0.6221 | 0.191 | 0.0475 | 5.68E-05 | Novel:one-stage design |
| rs62526122 | 8 | 92769569 | a | g | 0.2707 | 0.1739 | 0.0557 | 0.001806 | Novel:one-stage design |
| rs1986971 | 8 | 10268736 | a | g | 0.7048 | 0.2632 | 0.051 | 2.49E-07 | Novel:two-stage design |
| rs4598218 | 8 | 129483956 | t | c | 0.614 | 0.1523 | 0.048 | 0.001523 | Novel:two-stage design |
| rs4129585 | 8 | 143312933 | a | c | 0.4438 | 0.1977 | 0.0467 | 2.30E-05 | Novel:two-stage design |
| rs1906672 | 8 | 38130025 | a | g | 0.2275 | 0.2644 | 0.055 | 1.51E-06 | Novel:two-stage design |
| rs6996733 | 8 | 60535824 | t | c | 0.8439 | 0.1904 | 0.0647 | 0.003269 | Novel:two-stage design |
| rs72688070 | 8 | 81393697 | t | c | 0.1714 | -0.1536 | 0.0621 | 0.01338 | Novel:two-stage design |
| rs62491354 | 8 | 9730663 | a | g | 0.1401 | 0.3376 | 0.0663 | 3.59E-07 | Novel:two-stage design |
| rs35783704 | 8 | 105966258 | a | g | 0.1092 | -0.5219 | 0.0773 | 1.50E-11 | Previously reported |
| rs2898290 | 8 | 11433909 | t | c | 0.4835 | 0.3419 | 0.0466 | 2.12E-13 | Previously reported |
| rs4841569 | 8 | 11452177 | a | g | 0.4123 | -0.3758 | 0.0511 | 1.94E-13 | Previously reported |
| rs6557876 | 8 | 25900675 | t | c | 0.2511 | -0.3667 | 0.0533 | 5.98E-12 | Previously reported |
| rs520015 | 9 | 211762 | c | g | 0.5144 | 0.2043 | 0.0456 | 7.60E-06 | Novel:one-stage design |
| rs9886665 | 9 | 22942770 | t | c | 0.2721 | 0.1887 | 0.0519 | 0.000277 | Novel:one-stage design |
| rs60191654 | 9 | 753648 | a | g | 0.8143 | -0.2311 | 0.0584 | 7.50E-05 | Novel:one-stage design |
| rs7023828 | 9 | 128498594 | t | c | 0.423 | -0.2466 | 0.0464 | 1.10E-07 | Novel:two-stage design |
| rs1891730 | 9 | 130309028 | t | c | 0.6198 | -0.1749 | 0.0479 | 0.000257 | Novel:two-stage design |
| rs184457 | 9 | 131940019 | a | g | 0.2995 | -0.1157 | 0.0498 | 0.02015 | Novel:two-stage design |
| rs28558845 | 9 | 4334791 | c | g | 0.1568 | -0.2472 | 0.0652 | 0.00015 | Novel:two-stage design |
| rs1332813 | 9 | 9350706 | t | c | 0.3515 | 0.1771 | 0.0472 | 0.000175 | Novel:two-stage design |
| rs7045409 | 9 | 95201540 | a | t | 0.3681 | -0.1498 | 0.0473 | 0.001553 | Novel:two-stage design |
| rs111245230 | 9 | 113169775 | t | c | 0.9662 | -0.6917 | 0.1299 | 9.99E-08 | Previously reported |
| rs11592107 | 10 | 122968964 | a | g | 0.3087 | 0.2721 | 0.0495 | 3.93E-08 | Novel:one-stage design |
| rs72834453 | 10 | 124235226 | t | g | 0.8742 | -0.2378 | 0.0712 | 0.000839 | Novel:one-stage design |
| rs3802517 | 10 | 28233469 | a | t | 0.4668 | 0.188 | 0.0456 | 3.80E-05 | Novel:one-stage design |
| rs11187142 | 10 | 94468685 | t | c | 0.1047 | 0.298 | 0.0763 | 9.34E-05 | Novel:one-stage design |
| rs11197813 | 10 | 118523933 | a | g | 0.7025 | -0.1612 | 0.0505 | 0.0014 | Novel:two-stage design |
| rs7912283 | 10 | 133773019 | a | g | 0.642 | -0.2008 | 0.0505 | 7.02E-05 | Novel:two-stage design |
| rs1133400 | 10 | 134459388 | a | g | 0.7954 | -0.307 | 0.0601 | 3.24E-07 | Novel:two-stage design |
| rs34130368 | 10 | 48411796 | t | g | 0.1197 | -0.1772 | 0.0816 | 0.02998 | Novel:two-stage design |
| rs56352451 | 10 | 5804865 | t | c | 0.1337 | 0.3049 | 0.0672 | 5.69E-06 | Novel:two-stage design |
| rs12572586 | 10 | 74751579 | t | c | 0.9383 | -0.4496 | 0.1012 | 8.86E-06 | Novel:two-stage design |
| rs112184198 | 10 | 102604514 | a | g | 0.1058 | -0.5331 | 0.0761 | 2.40E-12 | Previously reported |
| rs1004467 | 10 | 104594507 | a | g | 0.9028 | 0.8884 | 0.0785 | 1.08E-29 | Previously reported |
| rs11191548 | 10 | 104846178 | t | c | 0.9129 | 1.0233 | 0.0818 | 6.19E-36 | Previously reported |
| rs4746172 | 10 | 75855842 | t | c | 0.7348 | -0.1017 | 0.0528 | 0.0542 | Previously reported |
| rs932764 | 10 | 95895940 | a | g | 0.5561 | -0.3654 | 0.0467 | 4.84E-15 | Previously reported |
| rs10766533 | 11 | 19224677 | a | t | 0.7004 | 0.2572 | 0.0515 | 5.85E-07 | Novel:one-stage design |
| rs11031051 | 11 | 30355707 | a | c | 0.683 | -0.1902 | 0.0493 | 0.000116 | Novel:two-stage design |
| rs190194639 | 11 | 34068037 | t | c | 0.0823 | 0.3274 | 0.0862 | 0.000146 | Novel:two-stage design |
| rs1585453 | 11 | 46884713 | a | t | 0.8866 | -0.2449 | 0.0775 | 0.00157 | Novel:two-stage design |
| rs4385883 | 11 | 51539339 | a | t | 0.7047 | 0.2189 | 0.0566 | 0.000112 | Novel:two-stage design |
| rs4980515 | 11 | 63744609 | t | c | 0.504 | 0.227 | 0.0464 | 1.01E-06 | Novel:two-stage design |
| rs67976715 | 11 | 68023742 | c | g | 0.2282 | 0.2708 | 0.0555 | 1.04E-06 | Novel:two-stage design |
| rs10743086 | 11 | 8774923 | a | g | 0.2086 | -0.2193 | 0.0567 | 0.000111 | Novel:two-stage design |
| rs7129220 | 11 | 10350538 | a | g | 0.1233 | 0.3919 | 0.0724 | 6.28E-08 | Previously reported |
| rs1401454 | 11 | 16250183 | t | c | 0.3998 | 0.3365 | 0.0469 | 7.10E-13 | Previously reported |
| rs757081 | 11 | 17351683 | c | g | 0.6644 | -0.2958 | 0.0487 | 1.29E-09 | Previously reported |
| rs5219 | 11 | 17409572 | t | c | 0.3755 | 0.32 | 0.0471 | 1.12E-11 | Previously reported |
| rs661348 | 11 | 1905292 | t | c | 0.5632 | -0.3417 | 0.0502 | 9.56E-12 | Previously reported |
| rs217727 | 11 | 2016908 | a | g | 0.192 | 0.3626 | 0.061 | 2.85E-09 | Previously reported |
| rs11537751 | 11 | 47587452 | t | c | 0.0521 | 0.3936 | 0.1076 | 0.000256 | Previously reported |
| rs11229457 | 11 | 58207203 | t | c | 0.2144 | -0.2886 | 0.0563 | 2.97E-07 | Previously reported |
| rs3741378 | 11 | 65408937 | t | c | 0.1328 | -0.4169 | 0.0696 | 2.15E-09 | Previously reported |
| rs7927515 | 11 | 76125330 | a | c | 0.3455 | 0.1705 | 0.0488 | 0.000479 | Previously reported |
| rs117206641 | 12 | 133086888 | t | c | 0.1145 | 0.3348 | 0.0783 | 1.88E-05 | Novel:one-stage design |
| rs28621435 | 12 | 13860990 | a | g | 0.1187 | -0.3138 | 0.0729 | 1.69E-05 | Novel:one-stage design |
| rs4143175 | 12 | 67782397 | t | c | 0.239 | 0.3055 | 0.0533 | 9.90E-09 | Novel:one-stage design |
| rs5742643 | 12 | 102837863 | t | c | 0.2505 | -0.2603 | 0.0534 | 1.07E-06 | Novel:two-stage design |
| rs11112548 | 12 | 105871914 | a | t | 0.9558 | 0.5768 | 0.1203 | 1.64E-06 | Novel:two-stage design |
| rs11571376 | 12 | 1059556 | c | g | 0.7011 | -0.1164 | 0.0506 | 0.0215 | Novel:two-stage design |
| rs2024385 | 12 | 12888438 | a | t | 0.4186 | -0.243 | 0.0467 | 1.99E-07 | Novel:two-stage design |
| rs7976167 | 12 | 24210599 | t | c | 0.6893 | 0.1409 | 0.0489 | 0.003922 | Novel:two-stage design |
| rs10437954 | 12 | 58003922 | a | g | 0.9064 | -0.4326 | 0.0832 | 2.01E-07 | Novel:two-stage design |
| rs7963801 | 12 | 79685226 | t | c | 0.4129 | -0.2145 | 0.0482 | 8.45E-06 | Novel:two-stage design |
| rs10858966 | 12 | 90567026 | c | g | 0.3035 | 0.2024 | 0.05 | 5.12E-05 | Novel:two-stage design |
| rs2384550 | 12 | 115352731 | a | g | 0.3457 | -0.2748 | 0.0473 | 6.29E-09 | Previously reported |
| rs1126930 | 12 | 49399132 | c | g | 0.0343 | 0.5757 | 0.14 | 3.93E-05 | Previously reported |
| rs73099903 | 12 | 53440779 | t | c | 0.0794 | 0.4218 | 0.0878 | 1.56E-06 | Previously reported |
| rs7297416 | 12 | 54443090 | a | c | 0.6867 | 0.2816 | 0.05 | 1.84E-08 | Previously reported |
| rs2681492 | 12 | 90013089 | t | c | 0.8344 | 0.7729 | 0.0615 | 3.26E-36 | Previously reported |
| rs17249754 | 12 | 90060586 | a | g | 0.1637 | -0.8015 | 0.0619 | 2.16E-38 | Previously reported |
| rs2480171 | 13 | 21559858 | t | c | 0.1324 | 0.2057 | 0.0693 | 0.002978 | Novel:one-stage design |
| rs1331012 | 13 | 27115424 | t | g | 0.269 | 0.1514 | 0.051 | 0.002962 | Novel:one-stage design |
| rs4274337 | 13 | 41967193 | a | g | 0.177 | -0.33 | 0.0612 | 6.93E-08 | Novel:one-stage design |
| rs75961402 | 13 | 56398286 | a | g | 0.1516 | 0.2759 | 0.0635 | 1.40E-05 | Novel:one-stage design |
| rs606950 | 13 | 22298923 | a | g | 0.6176 | 0.1755 | 0.047 | 0.000186 | Novel:two-stage design |
| rs9532243 | 13 | 32191408 | a | c | 0.4797 | 0.2485 | 0.0452 | 3.89E-08 | Novel:two-stage design |
| rs73187288 | 13 | 42738672 | a | c | 0.8935 | -0.2492 | 0.0738 | 0.000731 | Novel:two-stage design |
| rs912434 | 13 | 47189928 | t | g | 0.7628 | 0.2107 | 0.0531 | 7.30E-05 | Novel:two-stage design |
| rs9526707 | 13 | 51489186 | a | g | 0.3166 | -0.2364 | 0.0492 | 1.56E-06 | Novel:two-stage design |
| rs78474310 | 13 | 73826901 | a | g | 0.9566 | -0.4412 | 0.1138 | 0.000106 | Novel:two-stage design |
| rs7988232 | 13 | 79808655 | a | g | 0.4146 | 0.1378 | 0.0463 | 0.002917 | Novel:two-stage design |
| rs3011549 | 13 | 113634937 | a | c | 0.2888 | 0.226 | 0.0539 | 2.78E-05 | Previously reported |
| rs63418562 | 13 | 30146201 | t | c | 0.7462 | -0.3846 | 0.0529 | 3.74E-13 | Previously reported |
| rs34983854 | 14 | 39858442 | a | g | 0.6064 | -0.2259 | 0.0463 | 1.05E-06 | Novel:one-stage design |
| rs8014182 | 14 | 103859962 | t | c | 0.1388 | -0.3218 | 0.0655 | 8.80E-07 | Novel:two-stage design |
| rs17115145 | 14 | 30122409 | t | c | 0.3909 | 0.1853 | 0.0462 | 6.08E-05 | Novel:two-stage design |
| rs72683923 | 14 | 50735947 | t | c | 0.9767 | 0.7823 | 0.1705 | 4.45E-06 | Novel:two-stage design |
| rs11623535 | 14 | 72462381 | a | g | 0.7393 | 0.1623 | 0.0513 | 0.001552 | Novel:two-stage design |
| rs11159091 | 14 | 75074316 | a | g | 0.4654 | 0.1973 | 0.046 | 1.78E-05 | Novel:two-stage design |
| rs9888615 | 14 | 53377540 | t | c | 0.2936 | -0.2356 | 0.0499 | 2.32E-06 | Previously reported |
| rs8016306 | 14 | 63928546 | a | g | 0.7931 | 0.1339 | 0.0554 | 0.01569 | Previously reported |
| rs4965529 | 15 | 100145224 | a | c | 0.1657 | -0.2802 | 0.0622 | 6.60E-06 | Novel:two-stage design |
| rs11634028 | 15 | 76276150 | a | t | 0.205 | 0.2356 | 0.059 | 6.49E-05 | Novel:two-stage design |
| rs3743157 | 15 | 85680532 | a | c | 0.1651 | 0.2069 | 0.0615 | 0.000766 | Novel:two-stage design |
| rs11632436 | 15 | 86295286 | c | g | 0.5045 | 0.1907 | 0.0458 | 3.07E-05 | Novel:two-stage design |
| rs35199222 | 15 | 81013037 | a | g | 0.4398 | 0.2436 | 0.0466 | 1.75E-07 | Previously reported |
| rs2759308 | 15 | 81016227 | a | g | 0.4758 | 0.2592 | 0.046 | 1.79E-08 | Previously reported |
| rs2379829 | 16 | 3538873 | c | g | 0.728 | -0.2143 | 0.0521 | 3.84E-05 | Novel:one-stage design |
| rs34941092 | 16 | 50550137 | a | g | 0.1491 | -0.302 | 0.0651 | 3.53E-06 | Novel:one-stage design |
| rs1012089 | 16 | 74171973 | c | g | 0.4758 | -0.1354 | 0.0456 | 0.002974 | Novel:one-stage design |
| rs3851018 | 16 | 86437811 | c | g | 0.5676 | 0.2224 | 0.0473 | 2.60E-06 | Novel:one-stage design |
| rs6540125 | 16 | 87993889 | t | g | 0.3501 | 0.1864 | 0.0475 | 8.75E-05 | Novel:one-stage design |
| rs35450617 | 16 | 6889675 | t | g | 0.6958 | -0.1542 | 0.051 | 0.002489 | Novel:two-stage design |
| rs7187540 | 16 | 85318302 | a | c | 0.3245 | -0.193 | 0.0563 | 0.000606 | Novel:two-stage design |
| rs9899540 | 17 | 30777924 | a | t | 0.4126 | 0.1809 | 0.0487 | 0.0002 | Novel:one-stage design |
| rs112260610 | 17 | 64252393 | t | c | 0.1353 | 0.3389 | 0.0669 | 4.11E-07 | Novel:one-stage design |
| rs4925159 | 17 | 18185510 | a | g | 0.4192 | 0.2134 | 0.0464 | 4.23E-06 | Novel:two-stage design |
| rs1551355 | 17 | 30032420 | t | c | 0.2369 | 0.1842 | 0.0538 | 0.000621 | Novel:two-stage design |
| rs34430710 | 17 | 56876627 | a | t | 0.6753 | -0.2151 | 0.0487 | 9.87E-06 | Novel:two-stage design |
| rs1036902 | 17 | 58950791 | t | c | 0.8404 | -0.2107 | 0.0634 | 0.000888 | Novel:two-stage design |
| rs112280096 | 17 | 79367409 | a | c | 0.37 | -0.0932 | 0.0561 | 0.09643 | Novel:two-stage design |
| rs12946454 | 17 | 43208121 | a | t | 0.739 | -0.3193 | 0.0518 | 7.30E-10 | Previously reported |
| rs7406910 | 17 | 46688256 | t | c | 0.0893 | -0.4877 | 0.0812 | 1.93E-09 | Previously reported |
| rs8068318 | 17 | 59483766 | t | c | 0.7271 | 0.4318 | 0.0536 | 8.20E-16 | Previously reported |
| rs2240736 | 17 | 59485393 | t | c | 0.7328 | 0.4265 | 0.0525 | 4.49E-16 | Previously reported |
| rs1154214 | 18 | 24546824 | t | g | 0.3963 | -0.2163 | 0.046 | 2.57E-06 | Novel:one-stage design |
| rs6567160 | 18 | 57829135 | t | c | 0.7644 | 0.1618 | 0.0541 | 0.002765 | Novel:one-stage design |
| rs10460108 | 18 | 73034151 | a | g | 0.4819 | 0.2039 | 0.0452 | 6.40E-06 | Novel:one-stage design |
| rs11876341 | 18 | 48799991 | a | g | 0.6949 | -0.2167 | 0.0518 | 2.89E-05 | Novel:two-stage design |
| rs10048404 | 18 | 54578482 | t | c | 0.3741 | -0.2123 | 0.049 | 1.46E-05 | Novel:two-stage design |
| rs12454712 | 18 | 60845884 | t | c | 0.6224 | 0.1891 | 0.0537 | 0.000429 | Novel:two-stage design |
| rs34413141 | 18 | 777282 | a | t | 0.1796 | -0.337 | 0.0599 | 1.83E-08 | Novel:two-stage design |
| rs12958173 | 18 | 42141977 | a | c | 0.3 | 0.3518 | 0.0495 | 1.21E-12 | Previously reported |
| rs7256564 | 19 | 33889593 | a | g | 0.3133 | 0.2039 | 0.0487 | 2.87E-05 | Novel:one-stage design |
| rs73046792 | 19 | 49605705 | a | g | 0.1513 | -0.2413 | 0.069 | 0.000474 | Novel:one-stage design |
| rs2613765 | 19 | 5066330 | a | g | 0.4768 | -0.1874 | 0.0455 | 3.85E-05 | Novel:two-stage design |
| rs138877676 | 19 | 50935809 | t | g | 0.0211 | -0.5482 | 0.2033 | 0.006999 | Novel:two-stage design |
| rs17638167 | 19 | 11584818 | t | c | 0.047 | -0.5228 | 0.1095 | 1.81E-06 | Previously reported |
| rs8105753 | 19 | 31927547 | a | c | 0.6255 | 0.1895 | 0.0487 | 9.88E-05 | Previously reported |
| rs4247374 | 19 | 7252756 | t | c | 0.1355 | -0.5063 | 0.0753 | 1.76E-11 | Previously reported |
| rs1764975 | 20 | 4101290 | a | t | 0.7894 | 0.2759 | 0.058 | 1.99E-06 | Novel:one-stage design |
| rs6021247 | 20 | 50108980 | a | g | 0.5289 | 0.1623 | 0.0453 | 0.000338 | Novel:two-stage design |
| rs6031435 | 20 | 42797358 | a | g | 0.5388 | -0.2268 | 0.0456 | 6.72E-07 | Previously reported |
| rs11701033 | 21 | 33788341 | c | g | 0.8169 | -0.2465 | 0.0592 | 3.18E-05 | Previously reported |
| rs9608690 | 22 | 28921347 | a | g | 0.0678 | -0.308 | 0.0912 | 0.000733 | Novel:one-stage design |
| rs28578714 | 22 | 50727921 | t | c | 0.6045 | 0.2346 | 0.0538 | 1.28E-05 | Novel:two-stage design |
| * Candidate SNPs selected from Evangelou et al)[16].  † Regression coefficient and corresponding standard error derived from International Consortium for Blood Pressure Genome-Wide Association Studies (ICBP). [14] | | | | | | | | | |

| Table S2: Characteristics of variants used to develop genetic risk score to determine genetic susceptibility of atrial fibrillation for each person. (Derived from Nielsen et al.)[17] | | | | | | | | | | | |
| --- | --- | --- | --- | --- | --- | --- | --- | --- | --- | --- | --- |
| SNP | Chromosome | Position (GRCh37) | Risk allele | Reference allele | Freq reference allele | Odds ratio [95% CI] | P-value for association with atrial fibrillation | Excluded due to LD with other variants or blood pressure variants | Genome-wide association with cardiovascular disease or risk factors  (based on the NHGRI-EBI Catalog of genome-wide association studies; P-value <5 × 10^−8^) | Genome-wide association with cardiovascular disease or risk factors  (based on the MRC IEU OpenGWAS database; P-value <5 × 10^−8^) |  |
| rs284277 | 1 | 10790797 | C | A | 0.383 | 1.04 [1.03-1.06] | 1.25E-09 |  | No | Blood pressure |  |
| rs7529220 | 1 | 22282619 | C | T | 0.847 | 1.06 [1.04-1.08] | 1.98E-10 |  | PR interval | Already excluded |  |
| rs2885697 | 1 | 41544279 | G | T | 0.352 | 1.04 [1.03-1.06] | 2.88E-10 |  | Lean body mass | Already excluded |  |
| rs11590635 | 1 | 49309764 | A | G | 0.024 | 1.16 [1.10-1.21] | 4.12E-09 |  | No | No |  |
| rs146518726 | 1 | 51535039 | A | G | 0.033 | 1.17 [1.13-1.22] | 8.27E-15 | Yes | Already excluded | Already excluded |  |
| rs1545300 | 1 | 112464004 | C | T | 0.691 | 1.06 [1.04-1.07] | 1.48E-14 |  | Early cardiac repolarization measurement | Already excluded |  |
| rs4073778 | 1 | 116297758 | A | C | 0.564 | 1.05 [1.04-1.06] | 4.96E-13 |  | Pulse pressure measurement | Already excluded |  |
| rs79187193 | 1 | 147255831 | G | A | 0.943 | 1.12 [1.09-1.16] | 3.15E-14 |  | No | No |  |
| rs11264280 | 1 | 154862952 | T | C | 0.333 | 1.14 [1.13-1.16] | 3.07E-79 |  | No | No |  |
| rs72700114 | 1 | 170193825 | C | G | 0.076 | 1.22 [1.19-1.26] | 3.29E-54 |  | No | No |  |
| rs10753933 | 1 | 203026214 | T | G | 0.448 | 1.06 [1.05-1.08] | 9.84E-20 |  | No | No |  |
| rs4951258 | 1 | 205691316 | A | G | 0.416 | 1.04 [1.02-1.05] | 2.10E-08 |  | No | Anthropometric measures |  |
| rs7578393 | 2 | 26165528 | T | C | 0.796 | 1.06 [1.05-1.08] | 2.42E-12 |  | No | No |  |
| rs11125871 | 2 | 61470126 | C | T | 0.605 | 1.04 [1.03-1.05] | 6.42E-09 |  | No | No |  |
| rs2540949 | 2 | 65284231 | A | T | 0.615 | 1.07 [1.05-1.08] | 2.95E-22 |  | [Type II diabetes mellitus](https://www.ebi.ac.uk/gwas/efotraits/EFO_0001360) | Already excluded |  |
| rs6747542 | 2 | 70106832 | T | C | 0.536 | 1.06 [1.04-1.07] | 1.10E-16 |  | No | No |  |
| rs72926475 | 2 | 86594487 | G | A | 0.877 | 1.07 [1.05-1.09] | 2.37E-11 |  | No | No |  |
| rs28387148 | 2 | 127433465 | T | C | 0.105 | 1.08 [1.05-1.10] | 6.25E-11 |  | No | No |  |
| rs67969609 | 2 | 145760353 | G | C | 0.071 | 1.07 [1.05-1.10] | 1.71E-08 |  | No | No |  |
| rs56181519 | 2 | 175555714 | C | T | 0.732 | 1.07 [1.05-1.08] | 6.46E-18 |  | No | Blood pressure |  |
| rs2288327 | 2 | 179411665 | G | A | 0.156 | 1.10 [1.08-1.12] | 7.26E-25 |  | No | No |  |
| rs3820888 | 2 | 201180023 | C | T | 0.392 | 1.07 [1.06-1.09] | 5.75E-24 | Yes | Already excluded | Already excluded |  |
| rs35544454 | 2 | 213266003 | A | T | 0.808 | 1.06 [1.04-1.08] | 1.10E-11 |  | No | No |  |
| rs7650482 | 3 | 12841804 | G | A | 0.64 | 1.07 [1.06-1.09] | 1.79E-24 |  | No | No |  |
| rs73041705 | 3 | 24463235 | T | C | 0.702 | 1.05 [1.03-1.06] | 1.55E-09 |  | No | No |  |
| rs6790396 | 3 | 38771925 | G | C | 0.596 | 1.06 [1.05-1.08] | 2.40E-20 |  | [P wave duration](https://www.ebi.ac.uk/gwas/efotraits/EFO_0005094) | Already excluded |  |
| rs34080181 | 3 | 66454191 | G | A | 0.621 | 1.05 [1.03-1.06] | 1.28E-10 |  | No | Anthropometric measures |  |
| rs17005647 | 3 | 69406181 | T | C | 0.364 | 1.04 [1.03-1.06] | 2.70E-09 |  | No | No |  |
| rs6771054 | 3 | 89489529 | T | C | 0.596 | 1.05 [1.03-1.06] | 2.42E-11 |  | No | No |  |
| rs10804493 | 3 | 111554426 | A | G | 0.651 | 1.06 [1.04-1.07] | 1.63E-15 |  | No | No |  |
| rs1278493 | 3 | 135814009 | G | A | 0.436 | 1.04 [1.03-1.05] | 8.77E-09 |  | Waist-hip ratio, BMI-adjusted waist-hip ratio | Already excluded |  |
| rs7612445 | 3 | 179172979 | T | G | 0.188 | 1.05 [1.03-1.07] | 4.81E-09 |  | Heart rate, resting heart rate | Already excluded |  |
| rs60902112 | 3 | 194800853 | T | C | 0.226 | 1.05 [1.03-1.06] | 1.72E-08 |  | No | No |  |
| rs1458038 | 4 | 81164723 | T | C | 0.309 | 1.04 [1.03-1.06] | 1.74E-09 |  | Mean arterial pressure, diastolic blood pressure, systolic blood pressure | Already excluded |  |
| rs10006327 | 4 | 103890980 | C | T | 0.49 | 1.04 [1.02-1.05] | 4.42E-08 | Yes | Already excluded | Already excluded |  |
| rs67249485 | 4 | 111699685 | T | A | 0.199 | 1.44 [1.42-1.46] | 7.32e-443 |  | No | Ischemic stroke |  |
| rs6829664 | 4 | 114448656 | G | A | 0.262 | 1.06 [1.04-1.07] | 1.92E-13 |  | No | No |  |
| rs10213171 | 4 | 148937537 | G | C | 0.061 | 1.10 [1.07-1.12] | 1.32E-11 |  | No | No |  |
| rs12648245 | 4 | 174641184 | T | C | 0.924 | 1.10 [1.07-1.12] | 3.45E-13 | Yes | Already excluded | Already excluded |  |
| rs6596717 | 5 | 106427609 | C | A | 0.395 | 1.04 [1.03-1.06] | 3.00E-09 |  | No | No |  |
| rs337705 | 5 | 113737062 | G | T | 0.375 | 1.06 [1.04-1.07] | 1.63E-16 |  | No | No |  |
| rs2012809 | 5 | 128190363 | G | A | 0.79 | 1.06 [1.04-1.08] | 4.92E-10 | Yes | Already excluded | Already excluded |  |
| rs2040862 | 5 | 137419989 | T | C | 0.178 | 1.11 [1.10-1.13] | 1.08E-35 |  | No | No |  |
| rs6580277 | 5 | 142818123 | G | A | 0.237 | 1.07 [1.05-1.09] | 1.64E-17 |  | No | No |  |
| rs12188351 | 5 | 168386089 | A | G | 0.056 | 1.09 [1.06-1.12] | 2.52E-09 |  | No | No |  |
| rs6891790 | 5 | 172670745 | G | T | 0.717 | 1.08 [1.06-1.09] | 4.53E-22 |  | No | No |  |
| rs73366713 | 6 | 16415751 | G | A | 0.86 | 1.11 [1.09-1.13] | 1.53E-25 |  | No | No |  |
| rs34969716 | 6 | 18210109 | A | G | 0.305 | 1.07 [1.06-1.09] | 1.60E-19 |  | No | No |  |
| rs3176326 | 6 | 36647289 | G | A | 0.802 | 1.06 [1.05-1.08] | 1.42E-13 |  | QRS duration, PR interval | Already excluded |  |
| rs2031522 | 6 | 87821501 | A | G | 0.624 | 1.04 [1.03-1.06] | 1.47E-10 |  | No | No |  |
| rs3951016 | 6 | 118559658 | A | T | 0.459 | 1.07 [1.05-1.08] | 2.15E-22 |  | QRS duration | Already excluded |  |
| rs13195459 | 6 | 122403559 | G | A | 0.638 | 1.06 [1.05-1.08] | 4.15E-19 |  | No | No |  |
| rs117984853 | 6 | 149399100 | T | G | 0.101 | 1.13 [1.10-1.16] | 1.34E-24 |  | No | No |  |
| rs55734480 | 7 | 14372009 | A | G | 0.249 | 1.06 [1.04-1.07] | 2.20E-12 |  | No | No |  |
| rs6462079 | 7 | 28415827 | A | G | 0.721 | 1.05 [1.03-1.06] | 8.79E-10 |  | No | No |  |
| rs35005436 | 7 | 74134911 | C | T | 0.155 | 1.06 [1.04-1.08] | 3.34E-10 |  | No | Vascular/heart problems |  |
| rs56201652 | 7 | 92278116 | G | A | 0.733 | 1.05 [1.04-1.07] | 1.74E-12 |  | No | Anthropometric measures |  |
| rs11773845 | 7 | 116191301 | A | C | 0.586 | 1.11 [1.10-1.13] | 2.39E-55 |  | [PR interval](https://www.ebi.ac.uk/gwas/efotraits/EFO_0004462) | Already excluded |  |
| rs55985730 | 7 | 128417044 | G | T | 0.06 | 1.09 [1.06-1.12] | 5.24E-09 |  | No | No |  |
| rs7789146 | 7 | 150661409 | G | A | 0.821 | 1.06 [1.04-1.08] | 2.12E-11 |  | No | Blood pressure |  |
| rs35620480 | 8 | 11499908 | C | A | 0.157 | 1.06 [1.04-1.07] | 5.15E-09 | Yes | Already excluded | Already excluded |  |
| rs7508 | 8 | 17913970 | A | G | 0.711 | 1.07 [1.06-1.09] | 1.69E-21 |  | No | Triglycerides |  |
| rs7834729 | 8 | 21821778 | G | T | 0.885 | 1.07 [1.05-1.09] | 3.55E-10 |  | No | No |  |
| rs62521286 | 8 | 124551975 | G | A | 0.066 | 1.13 [1.10-1.16] | 4.50E-19 |  | [Intraocular pressure measurement](https://www.ebi.ac.uk/gwas/efotraits/EFO_0004695) | Already excluded |  |
| rs6994744 | 8 | 141740868 | C | A | 0.495 | 1.04 [1.03-1.05] | 1.10E-09 |  | No | Blood pressure |  |
| rs10821415 | 9 | 97713459 | A | C | 0.413 | 1.09 [1.07-1.10] | 2.92E-34 |  | No | No |  |
| rs2274115 | 9 | 139094773 | G | A | 0.7 | 1.05 [1.03-1.07] | 1.69E-10 |  | No | No |  |
| rs12245149 | 10 | 65321147 | C | A | 0.526 | 1.05 [1.03-1.06] | 1.66E-12 |  | No | Triglycerides |  |
| rs7096385 | 10 | 69664881 | T | C | 0.092 | 1.07 [1.05-1.10] | 4.87E-08 |  | No | Blood pressure |  |
| rs60212594 | 10 | 75414344 | G | C | 0.856 | 1.12 [1.10-1.15] | 9.20E-35 |  | No | Blood pressure |  |
| rs10458660 | 10 | 77936576 | G | A | 0.173 | 1.06 [1.04-1.07] | 6.78E-10 |  | No | No |  |
| rs11598047 | 10 | 105342672 | G | A | 0.162 | 1.17 [1.15-1.19] | 8.95E-66 |  | No | No |  |
| rs10749053 | 10 | 112576695 | T | C | 0.158 | 1.06 [1.04-1.08] | 1.05E-08 |  | No | No |  |
| rs10741807 | 11 | 20011445 | T | C | 0.245 | 1.08 [1.06-1.09] | 1.59E-20 |  | No | No |  |
| rs4935786 | 11 | 121661507 | T | A | 0.267 | 1.05 [1.03-1.06] | 4.85E-09 |  | No | No |  |
| rs76097649 | 11 | 128764570 | A | G | 0.093 | 1.12 [1.10-1.15] | 1.26E-20 |  | No | No |  |
| rs4963776 | 12 | 24779491 | G | T | 0.818 | 1.10 [1.08-1.11] | 1.84E-25 |  | No | No |  |
| rs17380837 | 12 | 26345526 | C | T | 0.693 | 1.05 [1.04-1.07] | 4.80E-12 |  | No | No |  |
| rs12809354 | 12 | 32978437 | C | T | 0.144 | 1.07 [1.05-1.09] | 2.89E-14 |  | No | No |  |
| rs2860482 | 12 | 57105938 | A | C | 0.274 | 1.06 [1.04-1.07] | 1.21E-12 |  | No | Blood pressure |  |
| rs71454237 | 12 | 70013415 | G | A | 0.791 | 1.06 [1.05-1.08] | 1.78E-13 |  | No | No |  |
| rs12426679 | 12 | 76237987 | C | T | 0.472 | 1.04 [1.03-1.05] | 4.95E-09 |  | No | No |  |
| rs883079 | 12 | 114793240 | T | C | 0.707 | 1.10 [1.09-1.12] | 2.84E-40 |  | QRS complex, QRS duration, PR interval | Already excluded |  |
| rs10773657 | 12 | 123327900 | C | A | 0.138 | 1.06 [1.04-1.08] | 2.54E-08 |  | No | No |  |
| rs6560886 | 12 | 133150210 | C | T | 0.788 | 1.05 [1.03-1.07] | 1.49E-08 | Yes | Already excluded | Already excluded |  |
| rs9506925 | 13 | 23368943 | T | C | 0.267 | 1.05 [1.03-1.06] | 2.72E-09 |  | No | No |  |
| rs35569628 | 13 | 113872712 | T | C | 0.777 | 1.05 [1.03-1.06] | 1.38E-08 |  | No | No |  |
| rs422068 | 14 | 23864804 | C | T | 0.349 | 1.04 [1.03-1.06] | 3.87E-10 |  | [Resting heart rate](https://www.ebi.ac.uk/gwas/efotraits/EFO_0004351) | Already excluded |  |
| rs11156751 | 14 | 32990437 | C | T | 0.285 | 1.07 [1.06-1.09] | 6.94E-21 | Yes | Already excluded | Already excluded |  |
| rs73241997 | 14 | 35173775 | T | C | 0.142 | 1.08 [1.06-1.10] | 2.94E-15 |  | No | No |  |
| rs2738413 | 14 | 64679960 | A | G | 0.495 | 1.08 [1.07-1.10] | 2.55E-31 |  | No | No |  |
| rs74884082 | 14 | 73249419 | C | T | 0.75 | 1.05 [1.03-1.07] | 3.48E-10 |  | No | No |  |
| rs10873298 | 14 | 77426525 | C | T | 0.366 | 1.04 [1.03-1.06] | 7.07E-09 |  | No | No |  |
| rs147301839 | 15 | 57924714 | C | A | 0.007 | 1.39 [1.26-1.55] | 1.93E-10 | Yes | Already excluded | Already excluded |  |
| rs7170477 | 15 | 64103777 | A | G | 0.304 | 1.04 [1.03-1.05] | 4.98E-08 |  | No | No |  |
| rs74022964 | 15 | 73677264 | T | C | 0.157 | 1.12 [1.10-1.14] | 3.51E-36 |  | No | No |  |
| rs12908004 | 15 | 80676925 | G | A | 0.164 | 1.08 [1.06-1.10] | 4.12E-16 |  | No | No |  |
| rs4965430 | 15 | 99268850 | C | G | 0.386 | 1.05 [1.03-1.06] | 1.26E-10 |  | [TPE interval measurement](https://www.ebi.ac.uk/gwas/efotraits/EFO_0004644) | Already excluded |  |
| rs140185678 | 16 | 2003016 | A | G | 0.035 | 1.18 [1.13-1.23] | 2.43E-14 |  | No | No |  |
| rs2359171 | 16 | 73053022 | A | T | 0.176 | 1.19 [1.17-1.21] | 4.65E-91 |  | No | Ischemic stroke |  |
| rs7225165 | 17 | 1309850 | G | A | 0.887 | 1.07 [1.04-1.09] | 3.20E-09 |  | No | No |  |
| rs9899183 | 17 | 7452977 | T | C | 0.714 | 1.05 [1.03-1.06] | 2.02E-09 |  | Smoking status measurement, Diastolic blood pressure, Systolic blood pressure | Already excluded |  |
| rs72811294 | 17 | 12618680 | G | C | 0.887 | 1.07 [1.05-1.10] | 9.67E-12 |  | No | No |  |
| rs11658278 | 17 | 38031164 | T | C | 0.479 | 1.05 [1.03-1.06] | 3.47E-11 |  | No | No |  |
| rs1563304 | 17 | 44874453 | T | C | 0.178 | 1.07 [1.05-1.09] | 2.56E-12 |  | No | No |  |
| rs12604076 | 17 | 76773638 | T | C | 0.478 | 1.04 [1.02-1.05] | 3.63E-08 |  | No | Blood pressure |  |
| rs9953366 | 18 | 46474192 | C | T | 0.663 | 1.05 [1.04-1.07] | 1.82E-11 |  | No | Anthropometric measures |  |
| rs8088085 | 18 | 48708548 | A | C | 0.535 | 1.04 [1.02-1.05] | 4.79E-08 |  | No | No |  |
| rs2834618 | 21 | 36119111 | T | G | 0.894 | 1.10 [1.08-1.12] | 3.41E-17 |  | No | No |  |
| rs464901 | 22 | 18597502 | T | C | 0.665 | 1.05 [1.04-1.07] | 1.53E-12 |  | No | No |  |
| rs133902 | 22 | 26164079 | T | C | 0.427 | 1.04 [1.03-1.06] | 9.14E-10 |  | No | No |  |

| Table S3: Summary of characteristics of 329,237 participants of white British ancestry included in the one-sample stratified Mendelian randomization. | | |
| --- | --- | --- |
| Characteristics | Atrial fibrillation cases  (n= 12,391) | Participant without atrial fibrillation  (n= 316,846) |
| Age (y), mean (SD) | 62.3 (5.8) | 56.7 (7.9) |
| Sex, n (%) |  |  |
| Men | 8,394 (5.5) | 143,102 (94.5) |
| Women | 3,997 (2.2) | 173,744 (97.8) |
| BMI (Kg/m^2^), mean (SD) | 28.9 (5.2) | 27.3 (4.6) |
| Smoking status, n (%) |  |  |
| Never | 5,453 (3) | 173,516 (97) |
| Previous | 5,718 (4.9) | 110,398 (95.1) |
| Current | 1156 (3.5) | 31853 (96.5) |
| Prefer not to answer | 64 (5.6) | 1079 (94.4) |
| Alcohol intake frequency, n (%) |  |  |
| Never | 1,089 (5.1) | 20,374 (94.9) |
| Special occasion only | 1,352 (3.9) | 33,536 (96.1) |
| 1-3 times a month | 1,168 (3.2) | 35,234 (96.8) |
| 1-2 times a week | 2,844 (3.3) | 83,897 (96.7) |
| 3-4 times a week | 2,804 (3.5) | 76,535 (96.5) |
| Daily or almost daily | 3,118 (4.4) | 67,070 (95.6) |
| Prefer not to answer | 16 (7.4) | 200 (92.6) |
| Systolic BP (mm Hg), mean (SD) | 141 (19.4) | 138.1 (18.5) |

| Table S4. Sensitivity analysis using a modified generic risk score for atrial fibrillation | | | | | | | | | |
| --- | --- | --- | --- | --- | --- | --- | --- | --- | --- |
| Categories of genetic susceptibility for AF | AF cases | Total | Prevalence of AF (%) | Mean and 95% confidence interval of observed SBP (mmHg) | Standard deviation of observed SBP (mmHg) | Beta (95% CI) * | Odds ratio (95% CI) † | p-value | p-trend |
| **Analysis using the main genetic risk score for AF (number of variants: 102)** | | | | | | | | | |
| Low | 1594 | 81520 | 1.95 | 138.29 (138.16 to 138.42) | 18.57 | 0.22 (0.21 to 0.24) | 1.20 (0.95 to 1.53) | 0.11 | < 0.001 |
| Mild | 2400 | 81519 | 2.94 | 138.33 (138.21 to 138.46) | 18.65 | 0.21 (0.20 to 0.23) | 1.34 (1.10 to 1.63) | 0.002 |  |
| Moderate | 3185 | 81519 | 3.90 | 138.22 (138.10 to 138.35) | 18.55 | 0.22 (0.21 to 0.24) | 1.43 (1.20 to 1.69) | < 0.001 |  |
| High | 5086 | 81520 | 6.23 | 138.10 (137.98 to 138.23) | 18.57 | 0.22 (0.21 to 0.24) | 1.51 (1.32 to 1.74) | < 0.001 |  |
| **Sensitivity analysis #1 (number of variants: 85)** | | | | | | | | | |
| Low | 1753 | 81519 | 2.15 | 138.24 (138.11 to 138.37) | 18.56 | 0.22 (0.21 to 0.23) | 1.16 (0.93 to 1.46) | 0.17 | < 0.001 |
| Mild | 2376 | 81520 | 2.91 | 138.39 (138.26 to 138.52) | 18.65 | 0.21 (0.20 to 0.23) | 1.43 (1.18 to 1.74) | < 0.001 |  |
| Moderate | 3192 | 81519 | 3.91 | 138.26 (138.13 to 138.39) | 18.58 | 0.23 (0.21 to 0.24) | 1.35 (1.14 to 1.60) | < 0.001 |  |
| High | 4941 | 81520 | 6.06 | 138.06 (137.93 to 138.19) | 18.55 | 0.22 (0.21 to 0.23) | 1.55 (1.34 to 1.78) | < 0.001 |  |
| **Sensitivity analysis #2 (number of variants: 68)** | | | | | | | | | |
| Low | 1914 | 81520 | 2.34 | 138.22 (138.09 to 138.34) | 18.55 | 0.21 (0.20 to 0.23) | 1.35 (1.09 to 1.68) | 0.005 |  |
| Mild | 2673 | 81519 | 3.27 | 138.35 (138.23 to 138.48) | 18.64 | 0.22 (0.20 to 0.23) | 1.17 (0.97 to 1.41) | 0.08 | < 0.001 |
| Moderate | 3219 | 81519 | 3.94 | 138.24 (138.11 to 138.36) | 18.59 | 0.24 (0.22 to 0.25) | 1.63 (1.38 to 1.94) | < 0.001 |  |
| High | 4459 | 81520 | 5.46 | 138.15 (138.02 to 138.27) | 18.56 | 0.22 (0.20 to 0.23) | 1.45 (1.23 to 1.67) | < 0.001 |  |
| SBP: systolic blood pressure; AF: atrial fibrillation  * Beta: regression coefficient for the association between SBP-genetic risk score (independent variable) and clinically measured SBP (dependent variable).  † Odds ratio per 10 mmHg genetically-predicted SBP  Sensitivity analysis #1: analysis using the main genetic risk score for AF, excluding variants associated with cardiovascular disease and risk factors (based on the NHGRI-EBI Catalog of genome-wide association studies, see Fig. S2. for the details)  Sensitivity analysis #2: analysis using the main genetic risk score for AF, excluding variants associated with cardiovascular disease and risk factors (based on the MRC IEU OpenGWAS database, see Fig. S2. for the details) | | | | | | | | | |

| Table S5: Characteristics of genetic variants used as proxies for classes of blood pressure-lowering drug effects. | | | | | | | | |
| --- | --- | --- | --- | --- | --- | --- | --- | --- |
| **ACEI** |  |  |  |  |  |  |  |  |
| SNP * | Position (GRCh37) | Allele1 | Allele2 | Freq1† | Effect† | Standard error† | P-value† | Sample size† |
| rs4291 | 17:61554194 | A | T | 0.6053 | -0.2836 | 0.0472 | 1.89E-09 | 287245 |
|  |  |  |  |  |  |  |  |  |
| **Beta-blocker** |  |  |  |  |  |  |  |  |
| SNP * | Position (GRCh37) | Allele1 | Allele2 | Freq1† | Effect† | Standard error† | P-value† | Sample size† |
| rs11196549 | 10:115707298 | A | G | 0.0421 | 0.7923 | 0.1255 | 2.75E-10 | 279594 |
| rs460718 | 10:115721364 | A | G | 0.3222 | -0.1832 | 0.0497 | 0.000226 | 279594 |
| rs11196597 | 10:115788094 | A | G | 0.1379 | 0.2439 | 0.0716 | 0.0006638 | 278589 |
| rs17875473 | 10:115800294 | T | C | 0.0944 | 0.2345 | 0.0844 | 0.005472 | 279595 |
| rs1801253 | 10:115805056 | C | G | 0.7305 | 0.4394 | 0.0524 | 5.08E-17 | 279594 |
| rs4359161 | 10:115826508 | A | G | 0.1822 | -0.2376 | 0.0592 | 6.09E-05 | 279593 |
|  |  |  |  |  |  |  |  |  |
| **Calcium channel blockers** |  |  |  |  |  |  |  |  |
| SNP * | Position (GRCh37) | Allele1 | Allele2 | Freq1† | Effect† | Standard error† | P-value† | Sample size† |
| rs3821843 | 10:18334521 | A | G | 0.2803 | 0.1453 | 0.0513 | 0.004658 | 279594 |
| rs114987861 | 10:18440444 | A | G | 0.3472 | 0.3351 | 0.0482 | 3.57E-12 | 277475 |
| rs113210396 | 10:18457722 | T | C | 0.9604 | -0.4955 | 0.1192 | 3.22E-05 | 278849 |
| rs7340705 | 10:18459450 | A | G | 0.7277 | -0.2133 | 0.0516 | 3.56E-05 | 278479 |
| rs2488136 | 10:18481737 | A | G | 0.911 | -0.1719 | 0.0865 | 0.0469 | 278588 |
| rs1888693 | 10:18514561 | T | C | 0.296 | 0.1356 | 0.0514 | 0.00836 | 270873 |
| rs16916914 | 10:18553968 | A | G | 0.5323 | -0.1507 | 0.0459 | 0.001027 | 279593 |
| rs7076319 | 10:18592450 | A | T | 0.7949 | 0.2464 | 0.061 | 5.32E-05 | 278589 |
| rs61278674 | 10:18627285 | A | G | 0.6157 | 0.143 | 0.0475 | 0.002622 | 279595 |
| rs1779209 | 10:18678987 | C | G | 0.1201 | 0.1752 | 0.072 | 0.01497 | 279592 |
| rs10828399 | 10:18695681 | T | C | 0.0266 | -0.4123 | 0.1724 | 0.0168 | 278594 |
| rs10828452 | 10:18710991 | A | G | 0.0234 | -0.6098 | 0.1781 | 0.0006184 | 273573 |
| rs10828542 | 10:18727901 | A | G | 0.7884 | -0.3403 | 0.0572 | 2.64E-09 | 278479 |
| rs12780039 | 10:18727959 | C | G | 0.7104 | 0.5426 | 0.0529 | 1.07E-24 | 278590 |
| rs112133583 | 10:18729855 | A | G | 0.0291 | -0.3976 | 0.1472 | 0.006913 | 278480 |
| rs11014170 | 10:18755664 | A | G | 0.7272 | -0.1349 | 0.0529 | 0.01072 | 268756 |
| rs7923191 | 10:18790727 | A | G | 0.5273 | 0.1149 | 0.0467 | 0.0138 | 271333 |
| rs12258967 | 12:2434419 | A | G | 0.6788 | 0.2237 | 0.0483 | 3.61E-06 | 287243 |
| rs72786098 | 12:2514270 | T | C | 0.2837 | 0.2199 | 0.0503 | 1.24E-05 | 287245 |
| rs1998822 | 12:49209340 | C | G | 0.0213 | 1.0616 | 0.1906 | 2.56E-08 | 272725 |
| rs4748474 | 3:53558012 | A | G | 0.6838 | 0.331 | 0.0524 | 2.62E-10 | 277474 |
| rs150857355 | 3:53605712 | A | G | 0.0305 | 0.395 | 0.1472 | 0.007298 | 278479 |
| rs2239046 | 3:53612327 | T | G | 0.0445 | -0.3563 | 0.1293 | 0.005856 | 278589 |
| rs714277 | 3:53734443 | T | C | 0.6684 | -0.1929 | 0.0485 | 7.08E-05 | 279594 |
| ACEI: angiotensin-converting enzyme inhibitors  * Candidate SNPs selected from Gill et al. [18].  † Regression coefficient and corresponding standard error derived from International Consortium for Blood Pressure Genome-Wide Association Studies (ICBP). [14] | | | | | | | | |
