# Supplementary material for: Genetic susceptibility, elevated blood pressure, and risk of atrial fibrillation: a Mendelian randomization study
Source: Genome Med. 2021 Mar 4;13:38. doi: 10.1186/s13073-021-00849-3 (PMC7934395; doi:10.1186/s13073-021-00849-3)
Supplement: Supplementary file 3 — Additional file 3. Figure S1. The genetic variant selection workflow for systolic blood pressure. Figure S2. The genetic variant selection workflow for atrial fibrillation. Figure S3. Distribution of atrial fibrillation genetic risk score created by 102 genetic variants on 329,237 participants of the UK Biobank with valid genetic data and full blood pressure measurement. Figure S4. Scatter plot of 254 genetic variants associated with systolic blood pressure and their effect on atrial fibrillation. Figure S5. Funnel plot of 254 variants, showing each variant causal estimate against instrument strength. Figure S6. The association between systolic blood pressure and risk of atrial fibrillation estimated by random-effect inverse variance weighted and applied various sensitivity analysis methods of two-sample Mendelian randomization. Figure S7. Leave-one-out plot to assess if a single genetic variant is driving the association between systolic blood pressure and atrial fibrillation. Figure S8. Sensitivity analysis for two-sample Mendelian randomization using a series of sequentially restricted linkage disequilibrium threshold for clumping. Figure S9. Multivariable Mendelian randomization results unadjusted and adjusted for body mass index to check the possibility of collider bias in association between systolic blood pressure and atrial fibrillation. Figure S10. One-sample Mendelian randomization for the association between systolic blood pressure per 10-mmHg and coronary heart disease and stroke as positive outcomes. Figure S11. One-sample Mendelian randomization for the association between systolic blood pressure per 10-mmHg and atrial fibrillation with the exclusion of all prevalent or incidence cases of coronary heart disease, heart failure and valvular heart disease. Figure S12. Sensitivity analysis for assessing the effect of case definition through ICD codes and self-report on the main estimation. Figure S13. Sensitivity analysis for assessing the effect of furthe [file 13073_2021_849_MOESM3_ESM.docx]

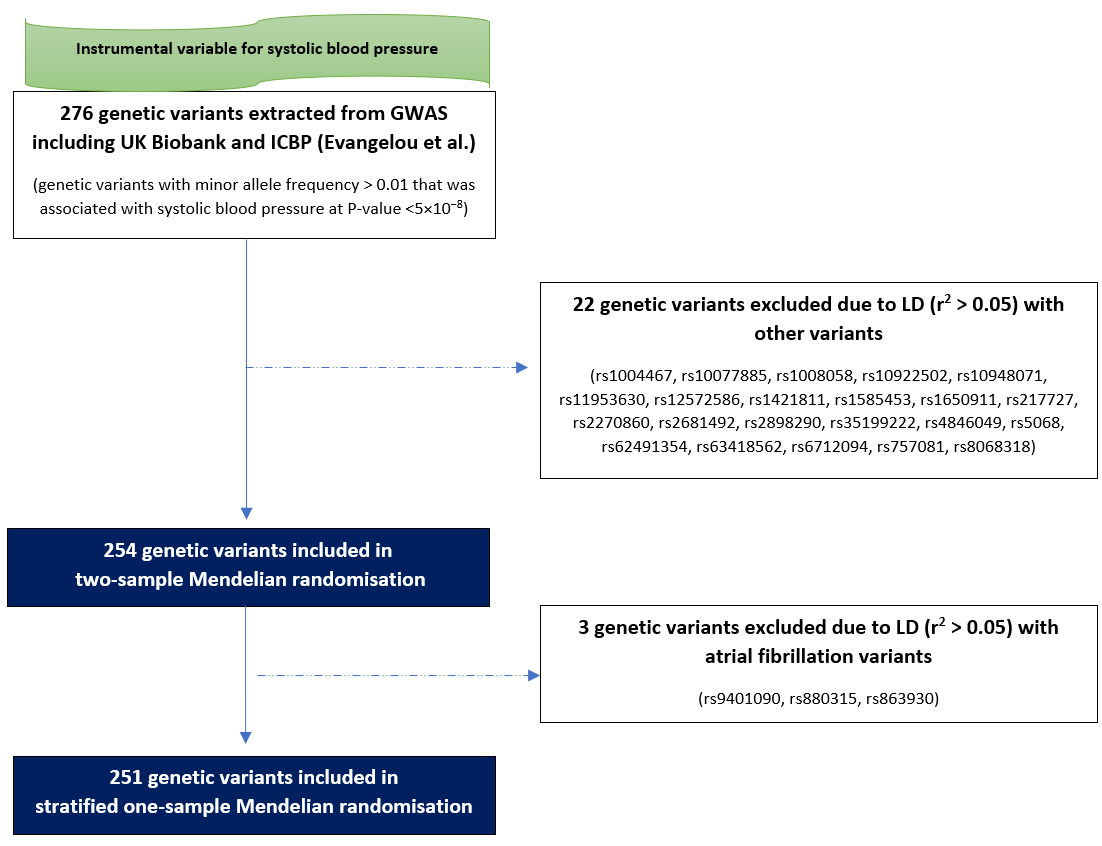


## Fig. S1. The genetic variant selection workflow for systolic blood pressure.

LD: linkage disequilibrium; GWAS: genome-wide association study


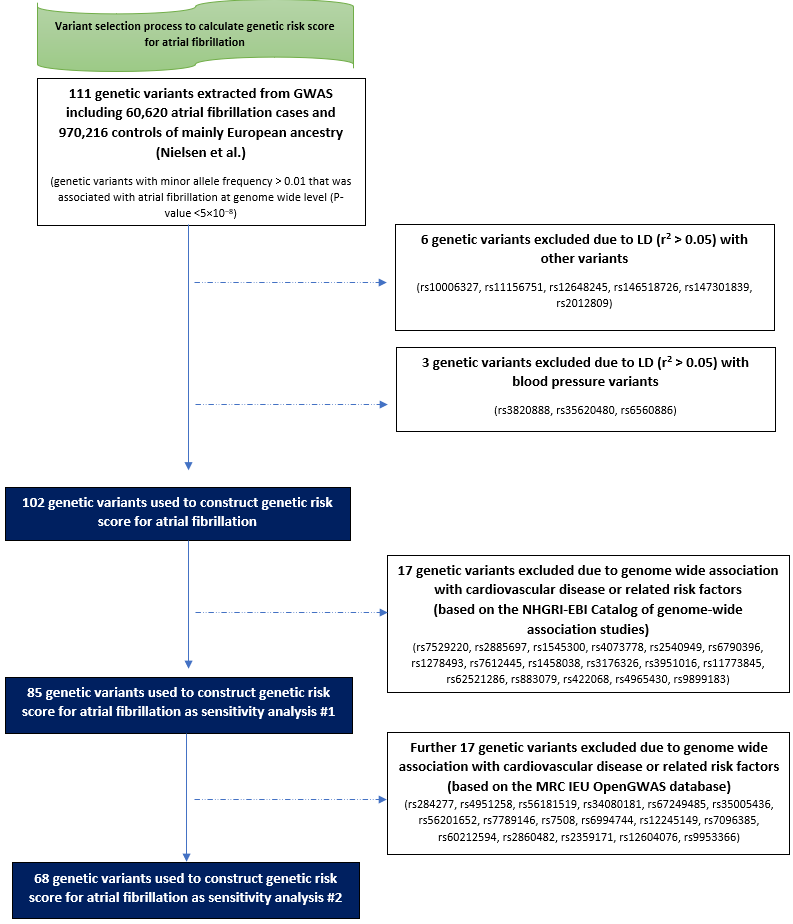


## Fig. S2. The genetic variant selection workflow for atrial fibrillation.

LD: linkage disequilibrium; GWAS: genome-wide association study


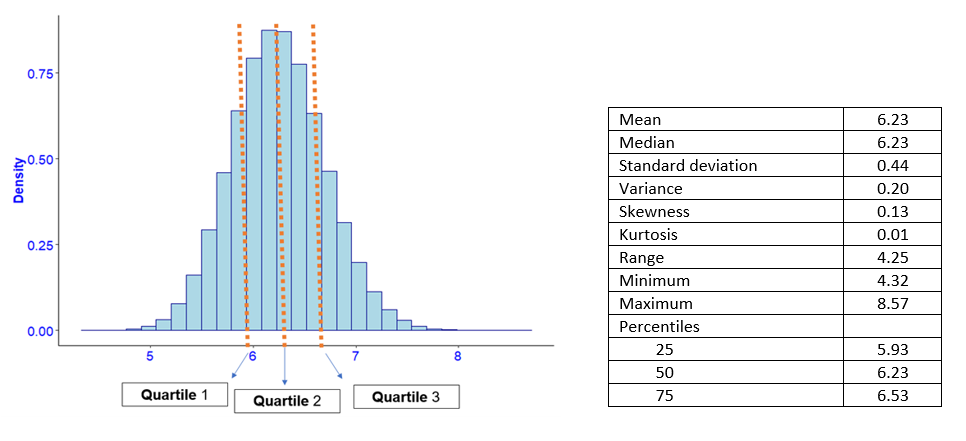


## Fig. S3. Distribution of atrial fibrillation genetic risk score created by 102 genetic variants on 329,237 participants of the UK Biobank with valid genetic data and full blood pressure measurement.


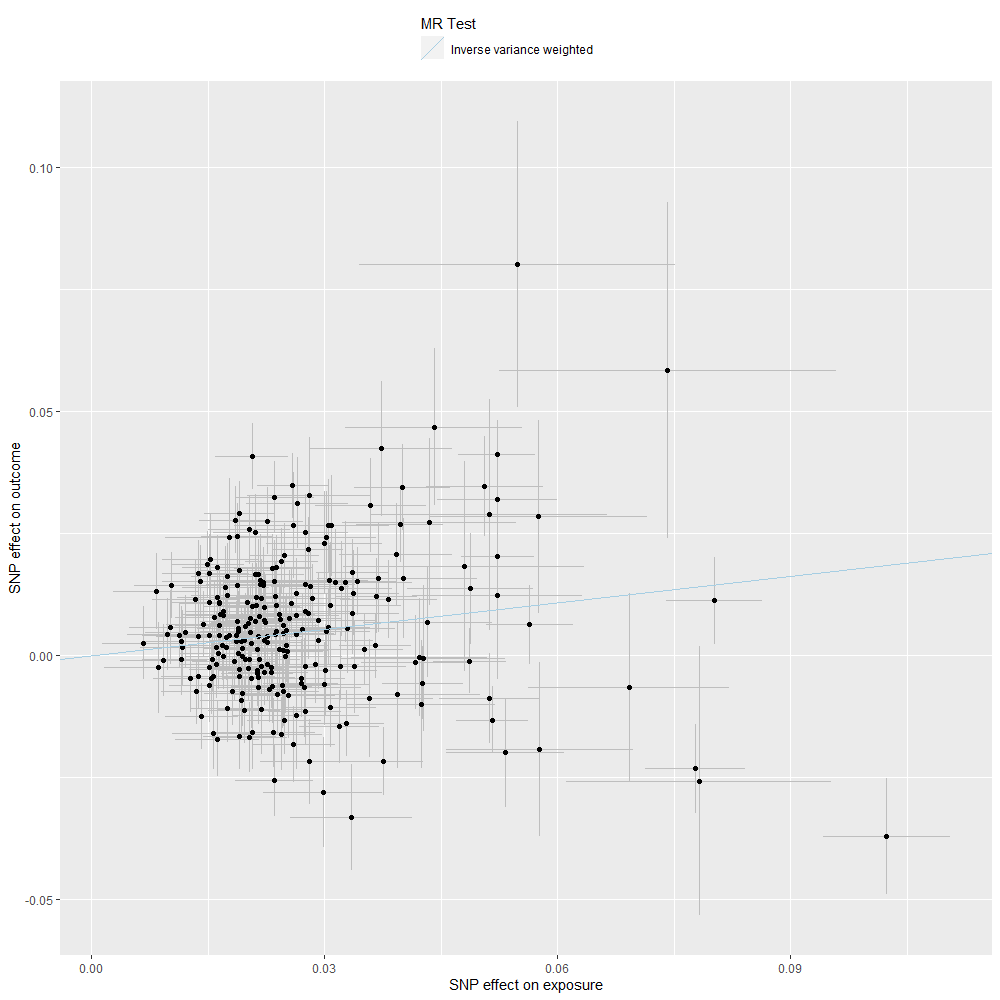


## Fig. S4. Scatter plot of 254 genetic variants associated with systolic blood pressure and their effect on atrial fibrillation.


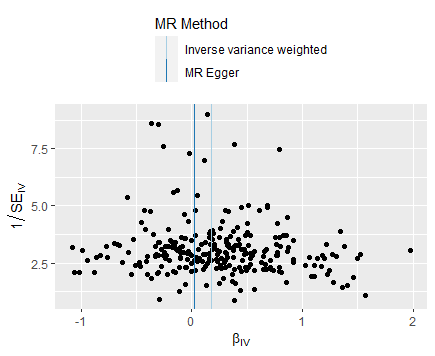


## Fig. S5. Funnel plot of 254 variants, showing each variant causal estimate against instrument strength.


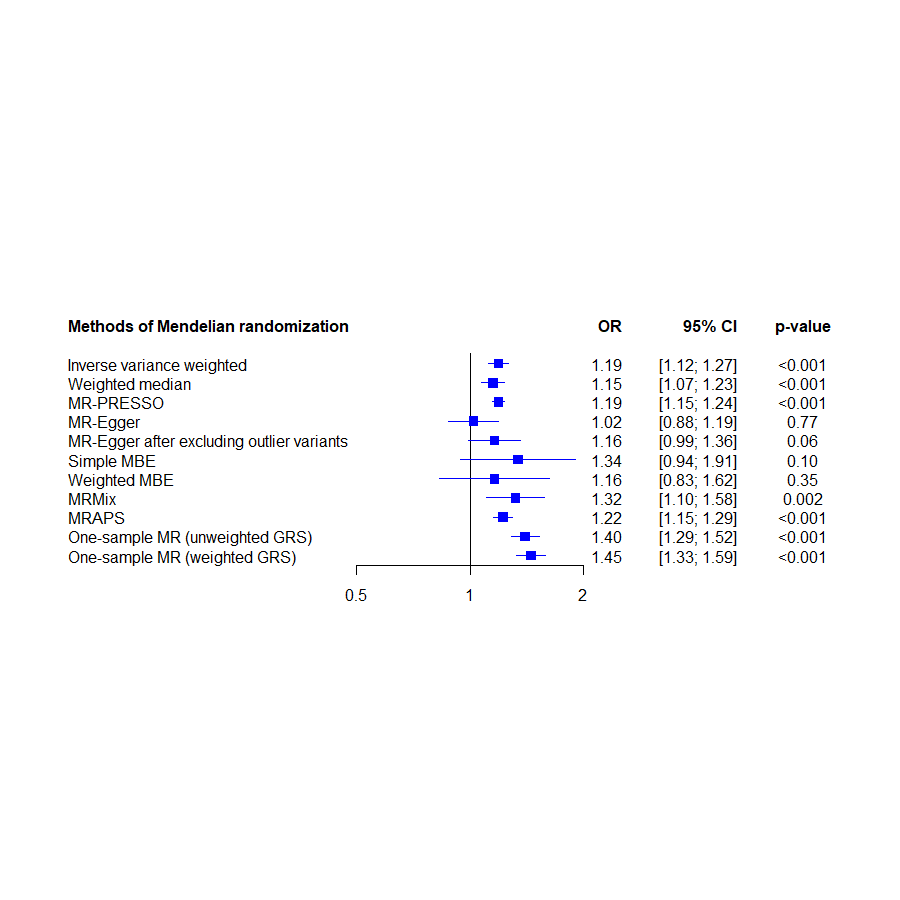


## Fig. S6. The association between systolic blood pressure and risk of atrial fibrillation estimated by random-effect inverse variance weighted and applied various sensitivity analysis methods of two-sample Mendelian randomization.

OR: odds ratio of atrial fibrillation per 10-mm Hg higher systolic blood pressure; CI: confidence interval; GRS: genetic risk score; RAPS: Robust Adjusted Profile Score; MBE: mode-based estimate; MR: Mendelian randomization; Cochran’s Q test for Inverse-variance weighted (heterogeneity test): 689.4, p-value < 0.001)


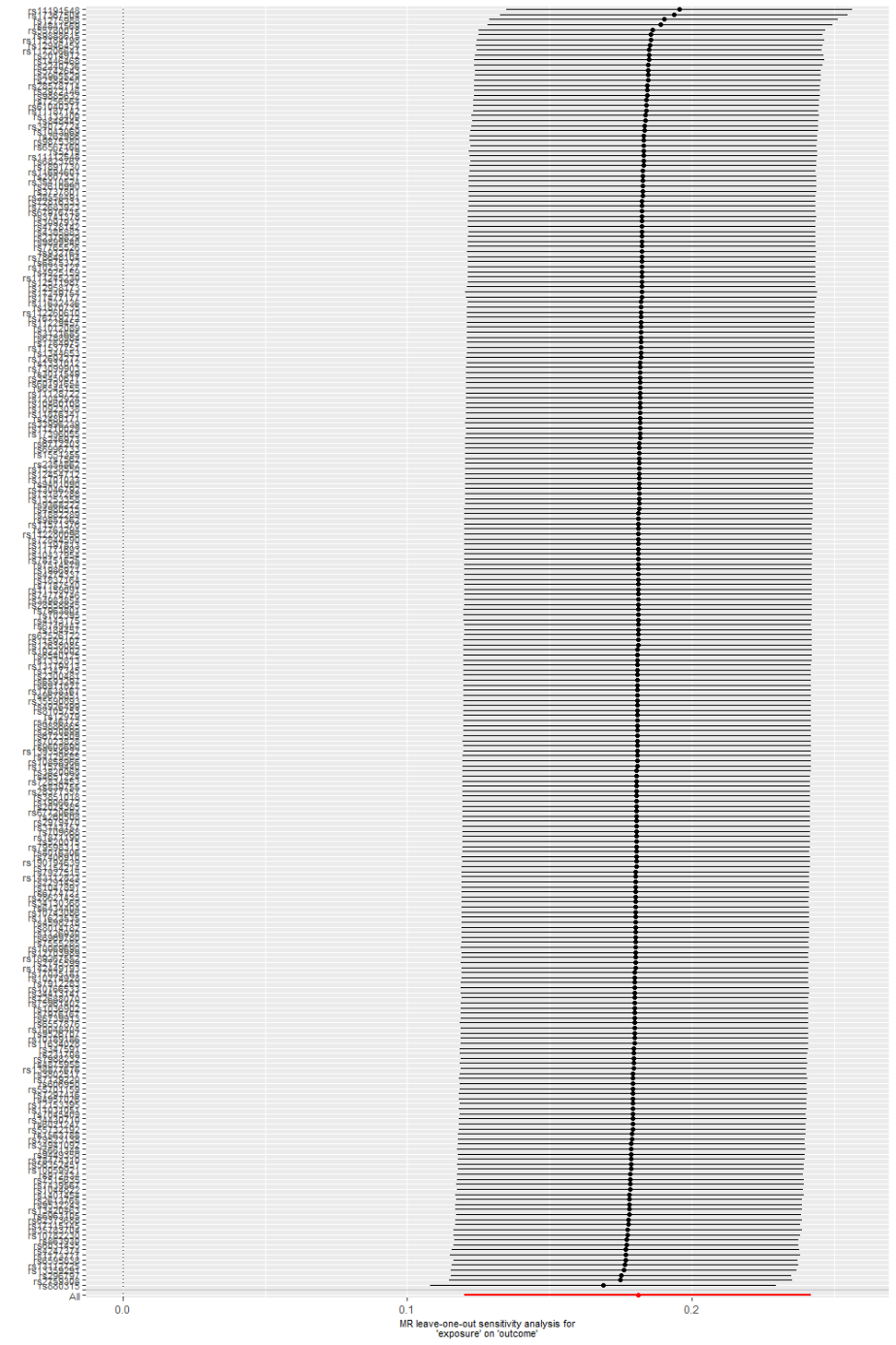


## Fig. S7. Leave-one-out plot to assess if a single genetic variant is driving the association between systolic blood pressure and atrial fibrillation.


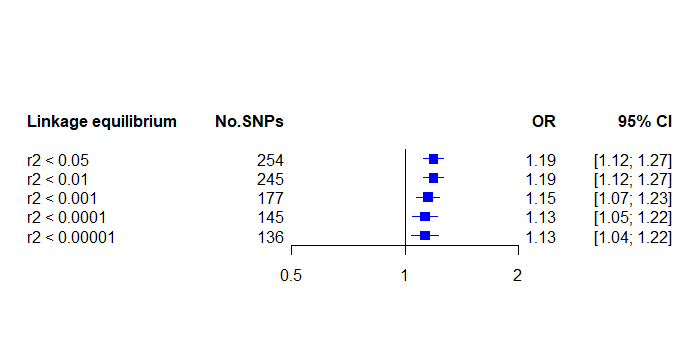


## Fig. S8. Sensitivity analysis for two-sample Mendelian randomization using a series of sequentially restricted linkage disequilibrium threshold for clumping.

OR: odds ratio of atrial fibrillation per 10-mm Hg higher systolic blood pressure; CI: confidence interval


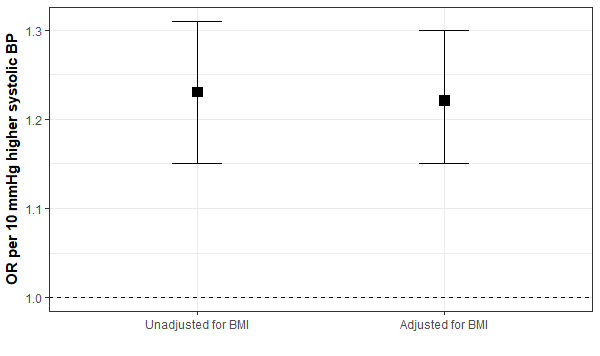


## Fig. S9. Multivariable Mendelian randomization results unadjusted and adjusted for body mass index to check the possibility of collider bias in association between systolic blood pressure and atrial fibrillation.


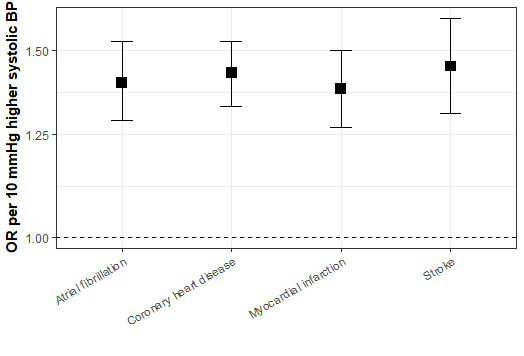


## Fig. S10. One-sample Mendelian randomization for the association between systolic blood pressure per 10-mmHg and coronary heart disease and stroke as positive outcomes.


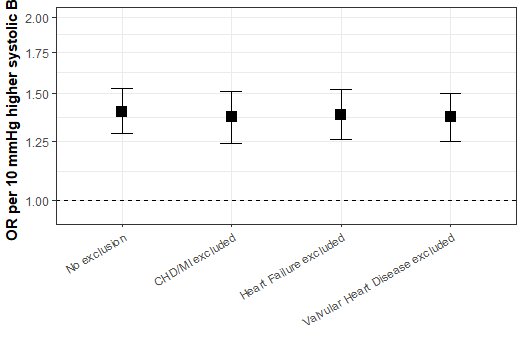


## Fig. S11. One-sample Mendelian randomization for the association between systolic blood pressure per 10-mmHg and atrial fibrillation with the exclusion of all prevalent or incidence cases of coronary heart disease, heart failure and valvular heart disease.


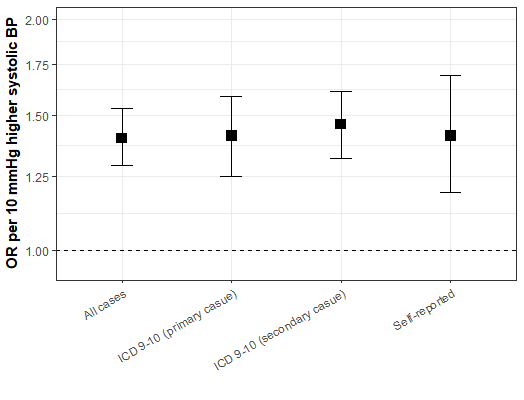


## Fig. S12. Sensitivity analysis for assessing the effect of case definition through ICD codes and self-report on the main estimation.

The wide confidence intervals in the analysis restricted to self-reported cases are due to the small number of atrial fibrillation patients diagnosed based on self-reported approach (number of self-reported patients: 2820 cases)


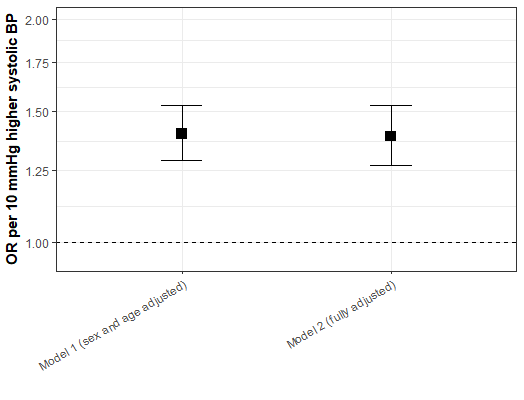


## Fig. S13. Sensitivity analysis for assessing the effect of further adjustment for well-known cardiovascular risk factors on the main estimation.

Model 1: adjusted for age and sex, UK Biobank assessment center, genotype measurement batch, genetic kinship to other participants and ten genetic principal components.

Model 2: adjusted for model 1, plus BMI, alcohol intake frequency, smoking status, Townsend deprivation index, LDL cholesterol level and blood glucose level.
